# Hydroxyhydroquinone as a novel modulator of mitochondrial metabolism with potential to restore CoQ10 homeostasis

**DOI:** 10.1007/s11010-026-05687-8

**Published:** 2026-08-07

**Authors:** Paulo Roberto dos Santos, Joseph Rastegar, Mariana Roesch-Ely

**Affiliations:** 1 Independent researcher, São Paulo, Brazil; 2 Institute of Biotechnology, University of Caxias do Sul, Caxias do Sul, 95070-560 Brazil

**Keywords:** Hydroxyhydroquinone, Ubiquinone, Coenzyme Q, Mitochondrial, Metabolism

## Abstract

Mitochondrial dysfunction is a hallmark of diverse metabolic and neurodegenerative disorders, often linked to impaired coenzyme Q_10_ (CoQ_10_) homeostasis. Here, we have evaluated the activity of hydroxyhydroquinone (HHQ) as a novel modulator of mitochondrial metabolism. Molecular simulations revealed that HHQ can act as an alternative aromatic substrate for human COQ2 in the CoQ_10_ biosynthetic pathway. In cultured cells, HHQ exposure (5.10^− 5^ mol.L^− 1^) enhanced complex I activity while maintaining stable ATP levels. HHQ reduced nitric oxide accumulation without altering superoxide dismutase activity, suggesting selective redox modulation. By bypassing the 4-hydroxybenzoic acid (PHBA) pathway, HHQ restores mitochondrial homeostasis and supports aerobic metabolism. These findings highlight HHQ as a small aromatic compound with strong redox potential that may favor metabolic functions driven by CoQ_10_ deficiency and mitochondrial dysfunction.

**Supplementary Information:**

The online version contains supplementary material available at https://doi.org/10.1007/s11010-026-05687-8.

## Introduction

Hydroxyhydroquinone (HHQ), or 1,2,4-trihydroxy benzene, is an aromatic molecule with an efficient redox potential, which can bind or release electrons according to the environment upon cellular metabolic demand [1]. Regardless of the variety of molecular targets that HHQ may play and interact with tissues and organs, it is known that HHQ participates actively in the mitochondrial energy metabolism [2]. HHQ can act as ubiquinol (CoQH_2_), a reduced form of Coenzyme Q (CoQ), and help transport electrons in the respiratory chain via the HHQ–HBQ (hydroxyhydroquinone–2-hydroxybenzoquinone) redox pair in the mitochondrial membrane. Coenzyme Q can also be found in a completely oxidized state (CoQ or ubiquinone), or in a semi-reduced state known as a semi-ubiquinone (CoQH⋅). It is well described that CoQ plays an essential role in the Electron Transport Chain (ETC), maintaining the electron flux from complexes I and II to complex III, which ultimately leads to a chemiosmotic gradient used to produce ATP by oxidative phosphorylation process [2, 3]. ETC represents one of the most complex redox biochemical systems found in cells. The components of this biochemical machinery present in all mitochondria operate in synchrony and with high specificity, which maintains a high conserved pathway across all eukaryotes.

The preserved regulatory mechanism of the biosynthesis pathway ultimately guaranties CoQ_10_ homeostasis [4]. CoQ_10_ is an essential element for mitochondrial function and provides antioxidant protection of cell membranes and plasma lipids [3]. The oxidation/reduction cycle of CoQ_10_ confers to this molecule the property to transfer electrons in different biological processes and to act as an antioxidant at cell membranes preserving lipids, protein, and nucleic acids from oxidative damage. Imbalances in either the biosynthesis pathway or in mitochondrial functions that impact CoQ_10_ homeostasis can lead to the development of various diseases, particularly those in which mitochondrial dysfunction plays a role [4, 5]. CoQ_10_ deficiency may affect tissues with higher CoQ_10_ levels and bioenergetic requirements. Generally, loss of homeostasis in CoQ_10_ pathway has been associated with age-related diseases [6], neurological disorders [7], kidney and liver diseases [8], and metabolic syndrome [3], among others.

CoQ_10_supplementation is commonly recommended for patients with mitochondrial diseases or secondary mitochondrial dysfunction. However, some special conditions such as genetic defects and poor bioavailability may limit the effectiveness of exogenous supplementations. CoQ levels can be secondarily decreased in a wide variety of mitochondrial diseases or can be reduced in a group of mitochondrial disorders such as primary CoQ deficiencies. Oral supplementation with high doses of CoQ_10_ is reported to show limited efficacy, particularly in patients with neurological symptoms [9]. This limitation maybe attributed to poor absorption and bioavailability of exogenous CoQ_10_, which restricts its access to affected tissues [10].

Literature has pointed out that exogenous CoQ_10_ treatment can be ineffective, most likely due to properties such as hydrophobicity and high molecular weight, generating poor solubility and bioavailability of CoQ_10_compared to shorter tail analogues like CoQ_4_. According to Steenberge, LH et al. (2023) [11], less hydrophobic CoQ species with shorter isoprenoid tails can serve as viable substitutes for CoQ_10_ in human cells. Their study demonstrate that CoQ_4_ can perform multiple functions of CoQ_10_ in CoQ-deficient cells at lower treatment concentrations, motivating further investigation of CoQ_4_ as a supplement for CoQ_10_ deficiencies.

Therefore, emerging therapies utilizing CoQ₁₀ analogs, biosynthetic precursors, and combination treatments, are emerging as alternatives to restore cellular energy. Novel therapeutic strategies employing structural analogs of the CoQ precursor 4-hydroxybenzoic acid alone or in combination are being developed. González-García, P et al. (2025) [10] investigated a combination of CoQ_10_ with vanillic acid, a structural analog of 4-hydroxybenzoic acid, and evaluated the effects in both murine and human models of primary CoQ deciency. The co-treatment revealed the liver displaying the most pronounced metabolic adaptations, restoring the expression of genes involved in sulfide oxidation and one-carbon metabolism pathways.

This paper identifies HHQ as a novel modulator of mitochondrial metabolism with potential to restore CoQ_10_ homeostasis. Strategies can improve mitochondrial and metabolic activities and decrease oxidative damage, one example is supplementation with CoQ_10_ [3, 5, 9]. In this complex scenario, HHQ arises as a metabolic alternative and powerful player that can bypass several steps in biosynthesis of CoQ_10_ pathway and reestablish homeostasis. In an analogous way, HHQ also plays an efficient redox potential accordingly to its concentration and bioavailability in different organisms. Literature has pointed tolerable doses of HHQ in rodents, from 0.1 g.L^− 1^ to 0.5 g.L^− 1^ [12] and in humans, from 0.1 to 0.7 mg.day^− 1^ [13] as a molecule present in roast green coffee beans [12, 14]. The LD50 from HHQ in rats is reported at doses between 0.35 and 0.5 g.kg^− 1^ of body weight for acute oral toxicity, with maximal non-lethal dose is 2 g.kg^− 1^ bw for dermal toxicity in rats [15]. HHQ shows promising potential in the fields of pharmacology and cosmetics, as it can effectively safeguard living cells from oxidative stress when administered at safe dosage levels [16, 17]. Most reports focus on the effects of HHQ present in roasted coffee in fat and vascular metabolism through redox balance. Studies have demonstrated that HHQ inhibits the antihypertensive effects of chlorogenic acid compounds found in coffee, both in rodents [18, 19] and humans [1, 14, 20, 21]. Reduction of fat catabolism after HHQ ingestion has also been investigated, although the exact mechanism of action remains unclear [12]. HHQ was able to decrease lipid catabolism in humans through ingestion of HHQ-reduced coffee, leading to significantly higher fat utilization in healthy humans [1]. From our previous in vitro studies, HHQ showed to have a strong redox potential at concentrations up to 5.10^− 5^ mol.L^− 1^ against insulin resistant cell line model (HepG2/IRM), acting as a reactive oxygen species (ROS) scavenger to ameliorate mitochondria activity and modulate ATP generation. ROS and ATP activities were maintained at relatively stable levels while great improvement in glucose uptake was observed after HHQ exposition to HepG2/IRM [22]. Free radicals often triggers a variety of diseases and research in literature suggests that they can have both positive and negative effects on health conditions, serving as regulators of cellular homeostasis [6, 23, 24]. A selective redox modulation is observed after HHQ exposition to cells and we believe that these events are strongly related with CoQ_10_ activity.

The biosynthesis of CoQ_10_ has been systematically investigated and occurs in a sequence of steps, where a series of membrane proteins positioned on the inner face of the mitochondrial matrix build the Coenzyme Q molecule and deliver it directly for use by the ETC [25]. The first enzyme in the series is a transmembrane protein called COQ2, a prenyltransferase of the UbiA superfamily that joins the substrates decaprenyl pyrophosphate and PHBA to form 3-decaprenyl-4-hydroxybenzoate. This initial intermediate is then transferred across the lipid membrane to subsequent enzymatic sites to be converted into CoQ_10_. Mutations in the human COQ2 gene [26] are responsible for a series of syndromes linked to CoQ_10_ production, among them neonatal type II diabetes mellitus and congenital cardiopathies [27, 28]. Specific mutations from a variety of UbiAs may lead to changes in substrate selectivity. Such changes are reported in literature and one example occurs in ApUbiA from the mushroom *Clitocybe clavipes* [29]. These studies have shown the key change in substrate selectivity after a mutation process, enabling this enzyme to transfer prenyl pyrophosphate chains to 1,4-hidroquinones (HQs), instead of PHBA. This phenomenon most likely changes selectivity of the enzymatic site of human homologous UbiA for the aromatic substrate, producing common CoQ_10_ intermediates to the known biochemical pathways.

**Fig. 1:**
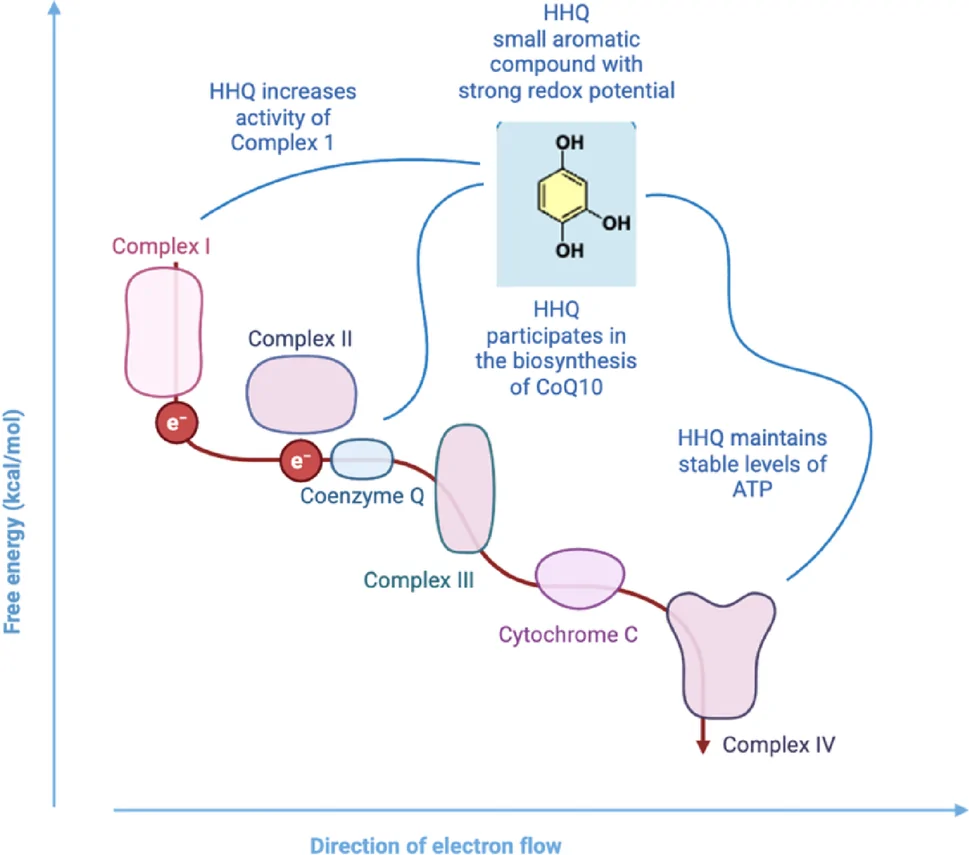
HHQ as a modulator of mitochondrial metabolism. A schematic image describing functions of HHQ inside the mitochondria compartment. HHQ participates in the biosynthesis of CoQ_10_ and interacts with the ETC in different manners, increasing complex 1 activity and maintaining ATP at stable levels

In the present study we highlight a schematic pathway of known mitochondrial COQ_10_ biosynthesis in mammals that starts with L-tyrosine to produce PHBA as aromatic building block. We bring a new pathway that bypasses the known model that begins with HHQ sharing some nonspecific COQ2 with PHBA on a key step of established model. Changes in substrate selectivity during biosynthesis of CoQ_10_ generate intermediates after HHQ exposure to cells, which stimulates complex 1 from the ETC while maintaining stable ATP levels. A schematic image describing functions of HHQ inside the mitochondria compartment is represented in Fig. 1.

It is known that hepatocytes and neurons perform the methylation of catechol groups via the catechol-O-methyltransferase (COMT) pathway to prevent their oxidation to 1,2-quinones, which are cytotoxic by inducing ROS formation [30]. Therefore, hepatocyte cultures tend to convert HHQ to 2-methoxy-1,4-hydroquinone (MHQ) as a protective mechanism. Another phenol derivatization mechanism is the sulfation of OH groups via the sulfotransferase (SULT) pathway [31]. This reaction is particularly important because it prevents the conversion of HHQ to 2-hydroxy-1,4-benzoquinone (HBQ), also cytotoxic due to reactive oxygen species production and its inhibition of the electron transport chain complex I [32].

Understanding HHQ as an acceptor of decaprenyl pyrophosphate chains in the biosynthesis of CoQ_10_ bring us towards a new potential metabolic route to be investigated, with novel molecular interactions that have not been described elsewhere. In this context, we investigated the hypothesis of HHQ, *O-*sulfated and 3-*O-*methylated derivatives metabolites integrating with the biosynthetic pathway of CoQ_10_ by assimilation through non-specific human COQ2 enzyme (*h*CoQ2). Therefore, we performed molecular docking simulations to evaluate the ability of HHQ likely derivative 2- methoxy-1,4-hydroquinone-4-sulfate (MHQ-4-sulfate) to act as aromatic substrates of *h*COQ2. A series of biochemical experiments on insulin resistant model cell line were also conducted to support our theory by assessing specific in vitro pathways of CoQ_10_, such as the activity of ETC complexes I and II, nitric oxide (⋅NO) generation, superoxide dismutase (SOD) activity, and ATP synthesis.

## Results

**Fig. 2:**
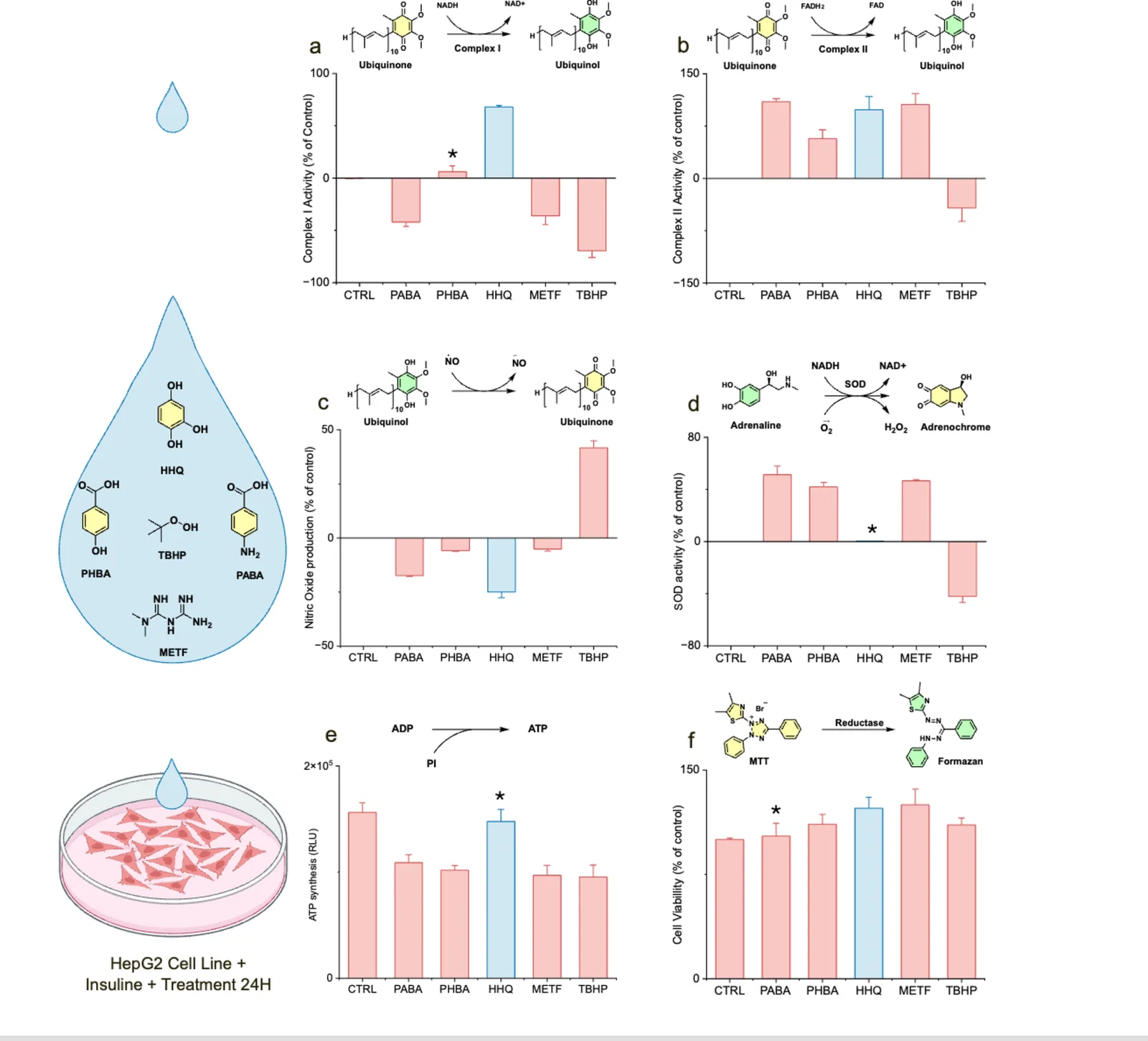
Results of experiments exposing cell cultures to treatments with different biochemical inducers in the presence of insulin (HepG2/IRM). Complex I of the electron transport chain (**a**); Complex II of the electron transport chain (**b**); nitric oxide (⋅NO) production (**c**); superoxide dismutase enzyme activity - SOD (**d**); ATP synthesis (**e**) and cell viability through MTT viability test (**f**). Control (CTRL), 4-aminobenzoic acid (PABA − 5.10^− 5^ mol.L^− 1^), 4-hydroxybenzoic acid (PHBA − 5.10^− 5^ mol.L^− 1^), Hydroxyhydroquinone (HHQ − 5.10^− 5^ mol.L^− 1^), Metformin (METF − 5.10^− 6^ mol.L^− 1^), Tert-Butyl Hydroperoxide (TBHP- 2.10^− 5^ mol.L^− 1^). The results were expressed as mean (bars) ± standard deviation (error bars) of three independent experiments performed in triplicate, used to assess normal distribution data. One-way analysis of variance (ANOVA) was applied, followed by Tukey post-hoc test. *p* ≤ 0.05. (*) no statistic difference to the control

All results of biochemical experiments exposing insulin resistant cell cultures (HepG2/IRM) to treatments with different biochemical inducers are summarized in Fig. 2: complex I and II of the electron transport chain, activity of nitric oxide, superoxide dismutase enzyme and ATP production. Cell viability through MTT was first performed to guarantee absence of cytotoxicity at all concentrations tested.

### Evaluation of the enzymatic activity of electron transport chain complex I, complex II

The enzymatic activity data of NADH: Ubiquinone reductase complex (Fig. 2a) revealed a generous increase in complex 1 activity in HHQ group compared to PHBA and control, while compounds PABA, METF, and TBHP presented characteristic of deactivators. It is noteworthy that complex I modulation was much more efficient in cells exposed to HHQ (+ 70% of control) compared to the known aromatic precursor for this purpose - PHBA (+ 10% of control), which also presented a slight positive shift in Complex I activity. PHBA showed a positive variation in activity but was not significant compared to the negative control. As for PABA, it showed a significant reduction in Complex I activity (−42% of control). It also revealed suppressive levels in METF group, most likely due to its insulin mimetic activity. TBHP was used as a positive control for oxidative stress induction, showing a suppressive effect of −69% compared to the negative control. In an interesting way, complex II activity showed similar patterns for HHQ, PABA and METF groups (+ 90% of control), with decrease levels for PHBA (+ 50% of control). TBHP was the only compounds that showed a reduction in Complex II activity (−40% of control) compared to all groups (Fig. 2b). Although HHQ is efficient in activating both complexes I and II, it plays a major function in the NADH ubiquinone reductase complex (C1). CoQ is reduced by NADH through Complex I activity, this cofactor selectively oxidizes NADH to NAD + ^31^.

### Nitric oxide (⋅NO), superoxide dismutase (SOD) and ATP activity

In the present study the HHQ group presented the greatest suppressive power on ⋅NO production, with a reduction of 25% compared to the negative control in the presence of insulin (Fig. 2c). ⋅NO levels were also suppressed for PABA, PHBA and METF, although in a less expressive manner. As expected, a great induction of ⋅NO was observed in the TBHP group (+ 50% of control). Despite HHQ presented the greatest inhibition of ⋅NO (−25% of control), HHQ treated group was not able to modulate SOD activity compared to the negative control. Compounds like PABA, PHBA, and METF exhibited significant increases in SOD activity after insulin intake, in the order of 51%, 42%, and 47%, respectively (Fig. 2d). Regardless of the precise mechanism of action, the results indicate that no overload of the antioxidant SOD defense complex was seen. It is worth noting that this experiment aimed not to determine the SOD enzyme expression, but to measure its activity through the production of the chromophore adrenochrome in vitro.

The positive control TBHP showed − 42% of SOD activity compared to the negative control, indicating a reduction in respiratory activity due to mitochondrial poisoning. These effects could be monitored both in the insulin resistance model and the cells without insulin. Since SOD is expressed in the mitochondrial matrix to capture the superoxide radical anion from Complex IV of the electron transport chain, the suppression of its activity due to poisoning of the catalytic site compromises mitochondrial defense.

We have recently investigated the in vitro ATP levels in cells using an insulin resistance model (HepG2/IRM) after HHQ exposition [22]. ATP activity from our previous results was monitored through a Luminescent Cell Viability Assay. This protocol allows determining the number of viable cells in culture based on the quantitation of ATP through the addition of luciferase, generating a luminescent signal. The results showed that ATP levels maintained stable after HHQ (5.10^− 5^ mol.L^− 1^) exposure in comparison to a significant decrease in ATP activity on HepG2/IRM treated with metformin (5.10^− 6^ mol.L^− 1^) and TBHP (2.10^− 5^ mol.L^− 1^). These results may indicate that the extra availability of glucose promoted by metformin can induce less profitable ATP pathways. In the present study we have monitored ATP through the Luminescent ATP for bioluminescence assay, using recombinant firefly luciferase and its substrate D-luciferin. The assay is based on luciferaseʼs requirement for ATP in producing light. ATP levels after HHQ exposition to cells were again consistent with our previous published results using other detection system, showing similar ATP levels from the HHQ group compared to the control. The other experimental conditions analyzed, such as the analogous PABA, PHBA, revealed significant lower ATP levels, compatible to the samples exposed to METF and TBHP (Fig. 2e).

Cell viability was monitored for all groups through MTT viability test (Fig. 2f). We also performed cell viability using a cytotoxicity detection kit method for quantifying cytotoxicity based on measuring the activity of the enzyme lactate dehydrogenase released from damaged cells. The results were consistent with the ones found for MTT that was used as a colorimetric assay measuring cellular metabolic activity to assess cell viability. The viability of cultures exposed to different treatments over a 24-hour interval was determined by measuring formazan production by mitochondrial activity using the MTT protocols [33]. The working concentrations obtained for the compounds HHQ, PABA and PHBA showed viabilities exceeding 100% of the control at concentrations of 5.10^− 5^ mol.L^− 1^; METF at 5.10^− 6^ mol.L^− 1^, and THBP at 2.10^− 5^ mol.L^− 1^. To establish an insulin-resistant cell model (HepG2/IRM), a gradient of insulin at different concentrations was initially tested under high glucose concentration (2.5.10^− 2^ mol.L^− 1^), as previous publications from our group [22]. Best parameters for insulin concentration was selected for insulin-resistance model and further experiments were processed with insulin at 5.10^− 6^ mol.L^− 1^ for 24 h. From the results published we could observe that glucose levels in cells after HHQ exposure revealed a significant increase compared to control. In addition, cells treated with METF and TBHP presented the lowest glucose uptake in comparison to control and HHQ [22].

### Metabolomic studies (LC-HRMS)

**Fig. 3:**
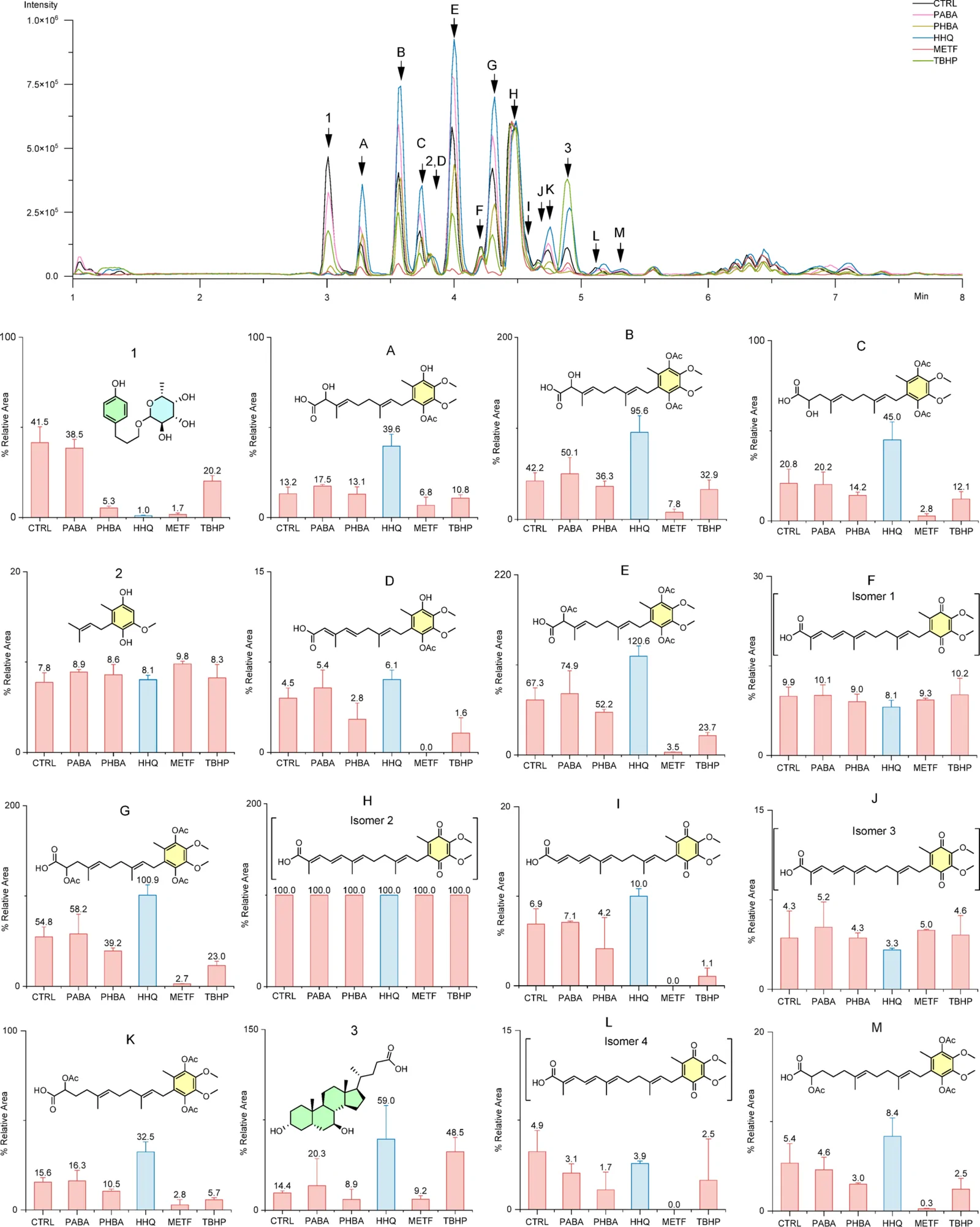
Overlay of Base Peak Chromatogram analysis of the six treatments (top). Extracted chromatographic area plots of 16 different metabolites detected at 2–6 min. Compound (H) was assign as an internal reference peak and showed a constant chromatographic area among all experiments. Note that 9 compounds (**A**, **B**, **C**, **D**, **E**, **G**, **I**, **K**, **M**) derived from ubiquinol presented an increase for HHQ on the extracted chromatographic area plots compared to all other groups. H- Internal reference peak. Data was acquired by two sets of 3 replicates, generating a pool of lysate for each sample analyzed. Extraction and treatment were processed withBruker Compass DataAnalysis software, data filters used were Base Peak Chromatogram of all MS, bbCID-BPC of all MS2, Extracted Ion Chromatogram (EIC) of all MS to integration areas

The cellular lysates obtained from the six treatments were analyzed by LC-ESI-HRMS to verify variations in the metabolomic profiles of the HEPG2/IRM cell line (Fig. 3; Table 1). The method allowed the identification of 13 catabolites of ubiquinol (A–M) originating from ω-oxidation of the side chain initiated by the CYP2E1 enzyme, followed by successive oxidations up to the third isoprenyl unit of ubiquinol [34, 35]. Compounds A, B, C, D, E, G, I, K and M derive from ubiquinol deactivated by 1,4 O-acetylation [36], whereas compounds F, H, J, and L are derived from ubiquinone. Compound H is the most intense peak in the control experiment (CTRL) and maintained a constant chromatographic area across all treatments, being assigned as the internal reference peak for comparative analyses (100%) for the other chromatographic peaks. Compounds F, H, J, and L were identified as a group of oxidized ubiquinone isomers. H states from internal reference peak used in the experiment. Table 1 brings the mass error in ppm of main metabolites identified on cell culture lysates. The largest mass error found was 3.2 for deoxycholic acid.

**Table 1:** Main metabolites identified on cell culture lysates

Compound	RT (min)	m/z	Molecular formula	Error (ppm)	Group	Ion
1	3.0	343.1395	C_15_H_23_O_8_	0.9	Tyrosine catabolite	[M+formate-H]^−^
A	3.3	421.1868	C_22_H_29_O_8_	0.1	CoQ_10_ catabolite	[M-H]^−^
B	3.6	463.1972	C_24_H_31_O_9_	0.3	CoQ_10_ catabolite	[M-H]^−^
C	3.7	477.2131	C_25_H_33_O_9_	0.1	CoQ_10_ catabolite	[M-H]^−^
2	3.8	221.1190	C_13_H_17_O_3_	0.0	Desmethoxy-CoQ_1_	[M-H]^−^
D	3.8	403.1770	C_22_H_27_O_7_	1.8	CoQ_10_ catabolite	[M-H]^−^
E	4.0	505.2085	C_26_H_33_O_10_	1.2	CoQ_10_ catabolite	[M-H]^−^
F	4.2	459.2029	C_25_H_31_O_8_	1.0	CoQ_10_ catabolite*	[M+formate-H]^−^
G	4.3	519.2249	C_27_H_35_O_10_	2.5	CoQ_10_ catabolite	[M-H]^−^
H	4.5	459.2033	C_25_H_31_O_8_	1.9	CoQ_10_ catabolite**	[M+formate-H]^−^
I	4.6	445.1878	C_24_H_29_O_8_	2.3	CoQ_10_ catabolite	[M-H]^−^
J	4.7	459.2029	C_25_H_31_O_8_	1.0	CoQ_10_ catabolite*	[M+formate-H]^−^
K	4.7	533.2408	C_28_H_37_O_10_	1.0	CoQ_10_ catabolite	[M-H]^−^
3	4.9	437.2923	C_25_H_41_O_6_	3.2	Deoxycholic acid	[M+formate-H]^−^
L	5.1	459.2028	C_25_H_31_O_8_	0.8	CoQ_10_ catabolite*	[M+formate-H]^−^
M	5.3	547.2524	C_29_H_39_O_10_	0.4	CoQ_10_ catabolite	[M-H]^−^

*Isomeric forms of C_25_H_31_O_8_

**Internal reference peak

An overlay of full Base Peak Chromatograms (BPC) (0–36 min) of 6 cell treatments obtained at negative ions polarity is represented in the supplementary material Fig. SI 1 (A) and an overlay of partial BPC chromatograms (2–8 min) of 6 cell treatments in Fig. SI 1 (B). Large peaks at 9.5, 18.0 and 28.0 min are background components of blank run performed with pure methanol. Fig. SI 1 (C) shows an overlay of Extracted Ion Chromatograms (EIC) of 14 major peaks of PABA treatment. Further data are highlighted in the Supplementary Information from Fig. SI 2 to Fig. SI 18. All mass spectra corresponding to the chromatographic peaks exhibited mass errors below 5.0 ppm relative to the calculated molecular formula (Table 1).

The treatment with HHQ activated both complex I and II, which brings information concerning variations in the catalytic activities of the respiratory chain (RC) I and II complexes. Such changes in cell metabolism may lead to increase in mitochondrial CoQ_10_concentration. Literature points out that CoQ_10_deactivation occurs through processes such as acetylation, phosphorylation, and sulfation [32, 36, 37], when the reduced form (CoQ_10_H_2_) reaches a threshold level, leading the deactivated conjugate to be metabolized via the oxidative pathway, which results in water-soluble compounds that are easily excreted.

**Fig. 4:**
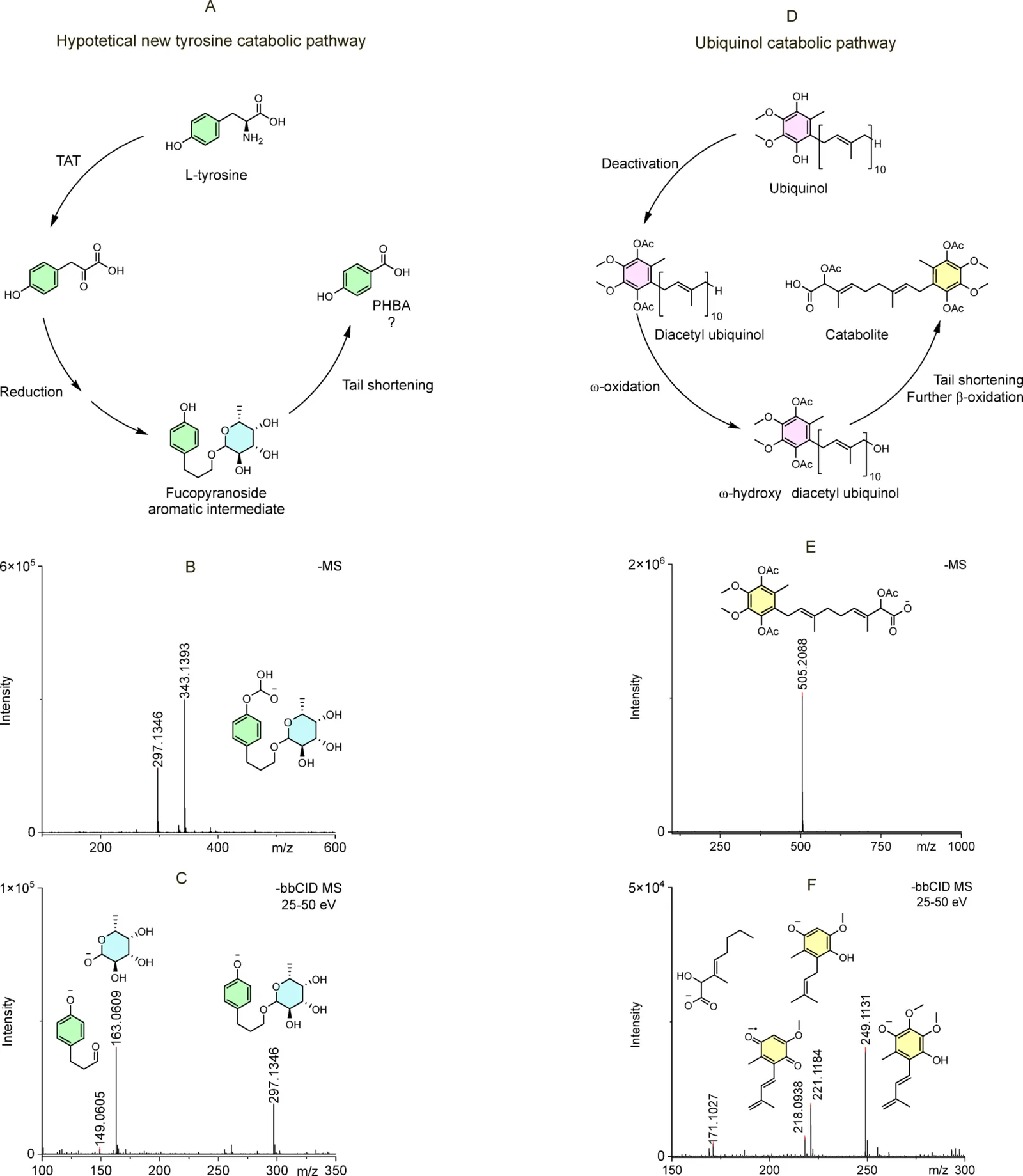
Proposed catabolic pathway of L-tyrosine to produce the hypothetical endogenous PHBA precursor (**A**). -MS spectrum (**B**) presents precursor ion at m/z 343.1393 attributed to form molecular adduct. Fragmentation spectrum (**C**) presents two major fragments correspondent to [M-H]- at m/z 297.1346 and m/z 163.0609 correspondent to fucose. Major CoQ_10_ catabolic pathway acting on HepG2 cell culture (**D**) and its major catabolite detected with m/z 505.2088 in 4.0 min (**E**). Fragmentation spectrum (**F**) presents four major fragments containing quinone ring (m/z 249.1131; 221.1194; 218.0938) and side chain fragment in m/z 171.1027

Compound 1 was identified as a probable tyrosine catabolism derivative, o-fucose-4-(3-hydroxypropyl)phenol (Fig. 3 (1). Fucosylation pathways are highly expressed in the HepG2 cell line, with recent studies employing GDP-fucose expression inhibitors to suppress liver tumor growth [38]. GDP-fucose is required for post-translational modifications involved in the production of glycoproteins, glycolipids, and oligosaccharides essential for cellular metabolism [39]. The reduced expression of the o-fucosylated derivative in HHQ and PHBA treatments suggests suppression of this fucosylation pathway, thereby inhibiting a probable PHBA production pathway. Suppression of the endogenous PHBA pathway is evident in HHQ and PHBA treatments, indicating that both aromatic compounds are required by the cell for CoQ_10_ production, as the cellular demand for substrates of the CoQ_10_ anabolic pathway was met by aromatic analogs. A proposed catabolic pathway from L-tyrosine to the hypothetical endogenous PHBA precursor is illustrated in Fig. 4.

Compound 2 was identified as desmethoxy CoQ1 (m/z 221.1183) and its expression remained without substantial change across all treatments (Fig. 3 (2)). Compound 3 was identified as deoxycholic acid, originating from the mitochondrial ω-oxidation of cholesterol, and is a common bile acid in hepatic metabolism [40] (Fig. 3 (3)). Treatments with HHQ and TBHP activate the biochemical pathway of deoxycholic acid, while PHBA, PABA, and METF reduce its expression below that of CTRL.

The compounds identified that were derived from ubiquinol after treatment with METF suppressed the O-acetylation and ω-oxidation pathways of ubiquinol to basal levels compared to CTRL. The result indicates an apparent suppression of mitochondrial activity and a consequent reduction in the cell’s oxygen demand. All the 9 compounds (A, B,C, D,E, G,I, K,M) derived from ubiquinol presented an increase for HHQ on the extracted chromatographic area plots compared to all other groups.

### Molecular docking simulations

UbiA *h*COQ2 is shown to be a non-selective enzyme [29, 41]. Prenylation reaction seems to be determined by thermodynamic tendency, following the Friedel-Crafts electrophilic substitution mechanism. Based on these premises, we conducted computational simulations to investigate the likely assimilation route of HHQ through the CoQ_10_ biosynthesis pathway (Fig. 4). In silico evaluations were processed to assess the interactions of HHQ metabolites competing with PHBA for the active site of the UbiA enzyme (COQ2). A potential prenylation of these compounds over -PHBA were also evaluated. The rigid docking results show that 2-methoxy-1,4-hidroquinone-4-sulfate (MHQ-4-sulfate) stabilize in the same cavity occupied by PHBA, and their positions seem to favor the interaction between cofactor and substrate. MHQ-4-sulfate is apparently more activated than PHBA and may react more readily at position C6 on aromatic ring (Fig. 4F).

**Fig. 5:**
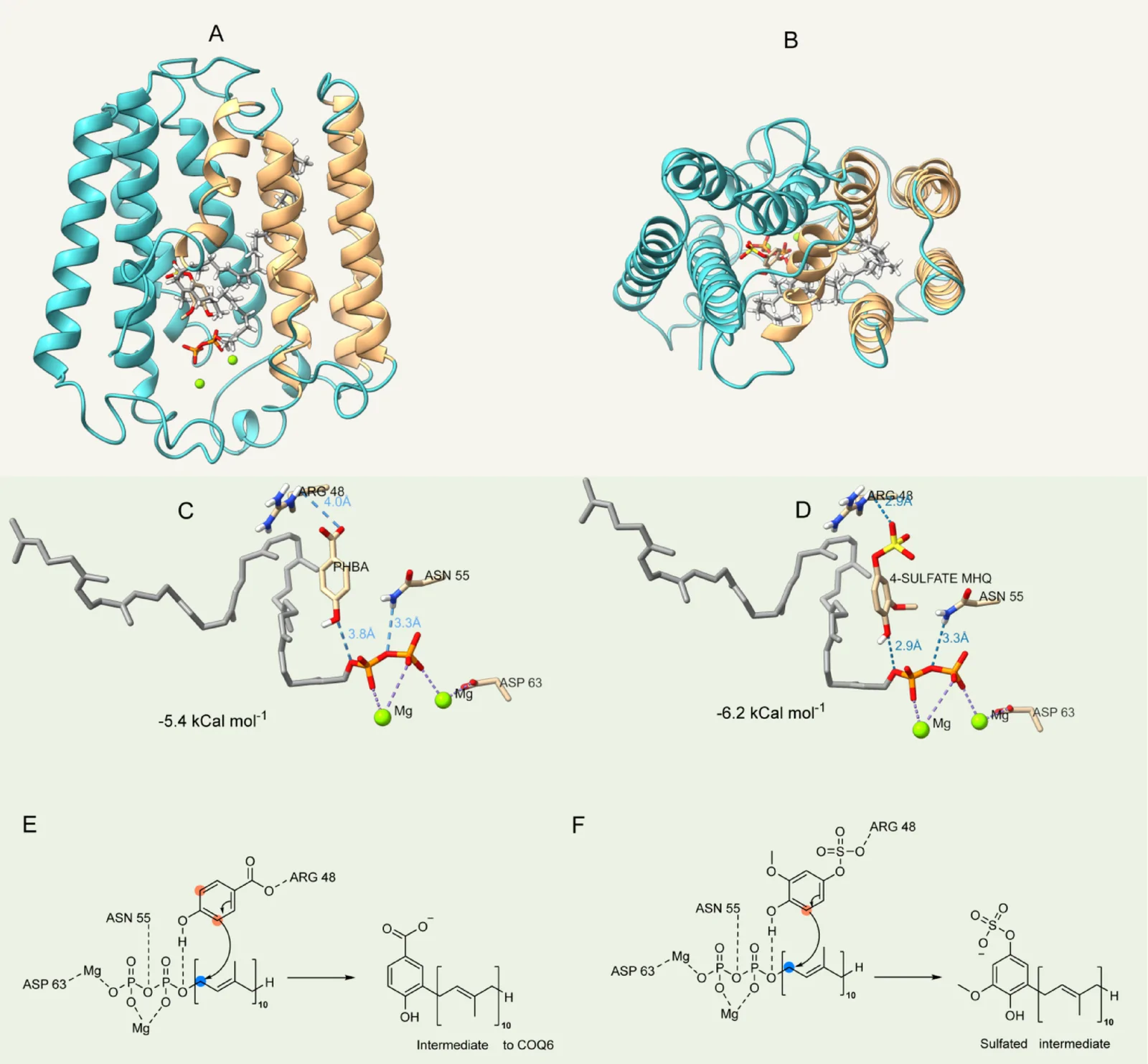
Docking experiment plots: the human COQ2 isoform 1 (hCOQ2) [23] were generated from Phyre2.2 server [70] using ApUbiA homolog of *Aeropyrum pernix* K1 (PDB 4OD5) as fold [71]. **A**, **B**) Cartoon structure of human *h*CoQ2 isoform 1 highlighting the access channel formed by helix S1, S6, S7, S8 and S9 in orange; **C**) The decaprenyl pyrophosphate substrate was kept in its active site bound to the two Mg^2+^ cations (pale green spheres). The MHQ-4-sulfate (**D**) model stabilize in positions similar to PHBA. Schematic overview of biochemical reacting sites for COQ2. **E**) Known COQ2 step by eukaryotes shows PHBA with two symmetrical position to react (carbon atom 2 and 6, in red); Proposed bypass pathway to produce COQ5 (**F**) substrate demanding mitochondrial aryl sulfatase

**Fig. 6:**
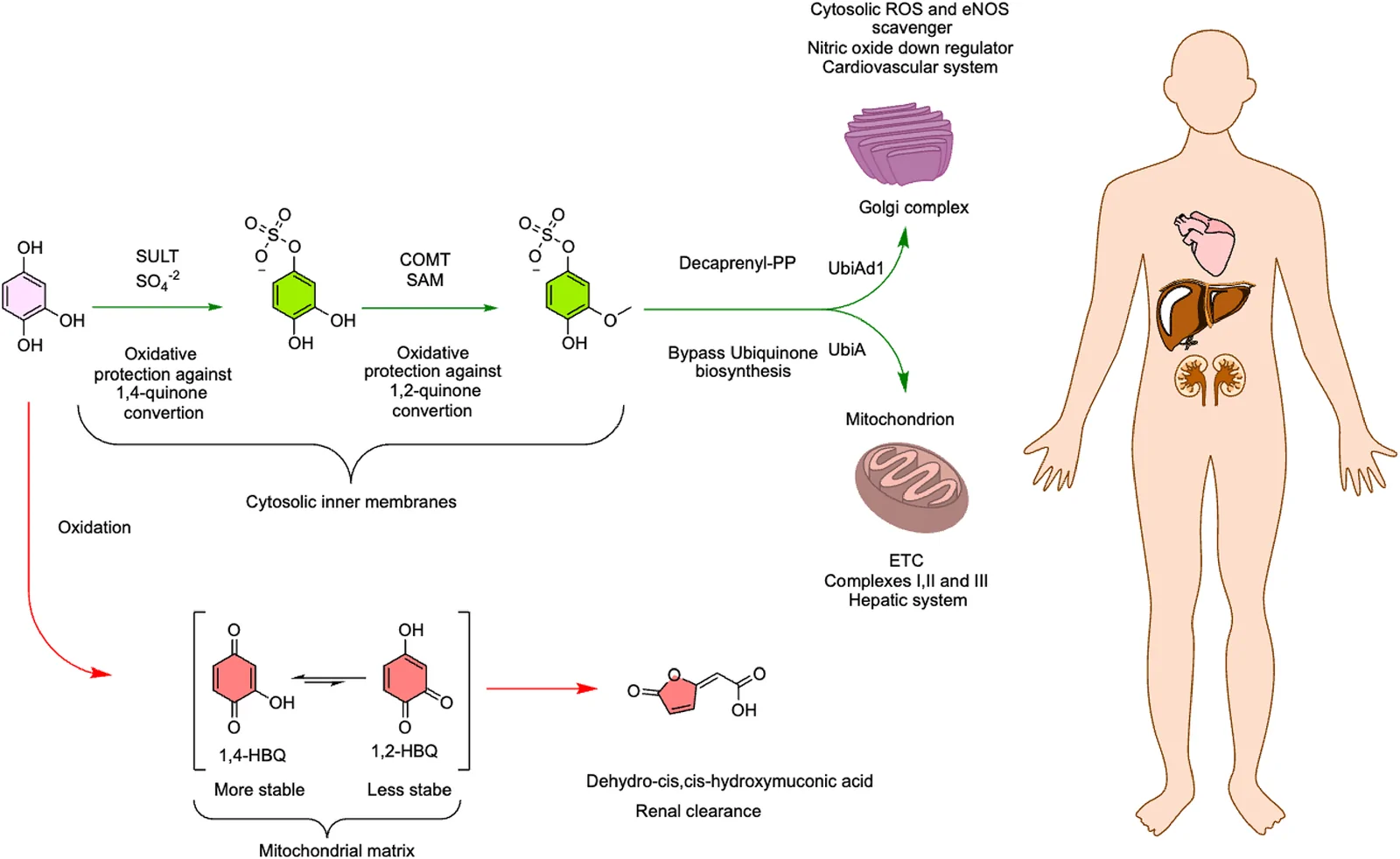
Mechanism of action proposed for HHQ, with anabolic and catabolic pathways. Anabolic (green arrows) leading to Golgi complex and/or bypassing ubiquinone biosynthesis. Catabolic pathway (red arrows) produces hydroxymuconic acid excreted by renal clearance

The choice of the molecular docking model used was based on known biochemical pathways [28]. It is known that *h*CoQ2 is a prenyltransferase of the UBiA superfamily that joins the substrates decaprenyl pyrophosphate and PHBA to form 3-decaprenyl-4-hydroxybenzoate. Literature brings classical chemical reacting sites for COQ2 by eukaryotes, where PHBA in shown in two symmetrical positions to react, C2 and C6 (Fig. 5E). Predictions of alternative docking poses are highlighted in the Fig. SI 19–21. A proposed bypass pathways to produce COQ5 substrate demanding mitochondrial aryl sulfatase (Fig. 5F) is compared to a bypass pathway proposed by Acosta, et al., (2019) [41] using vanillic acid as UbiA substrate to produce COQ4 intermediate. From the results presented, a mechanism of action is proposed for HHQ, with anabolic and catabolic pathways (Fig. 6). The anabolic is leading to Golgi complex and/or bypassing ubiquinone biosynthesis in the mitochondrial compartment. The catabolic pathway bellow produces hydroxymuconic acid, which may be excreted by renal clearance.

## Discussion

**Fig. 7:**
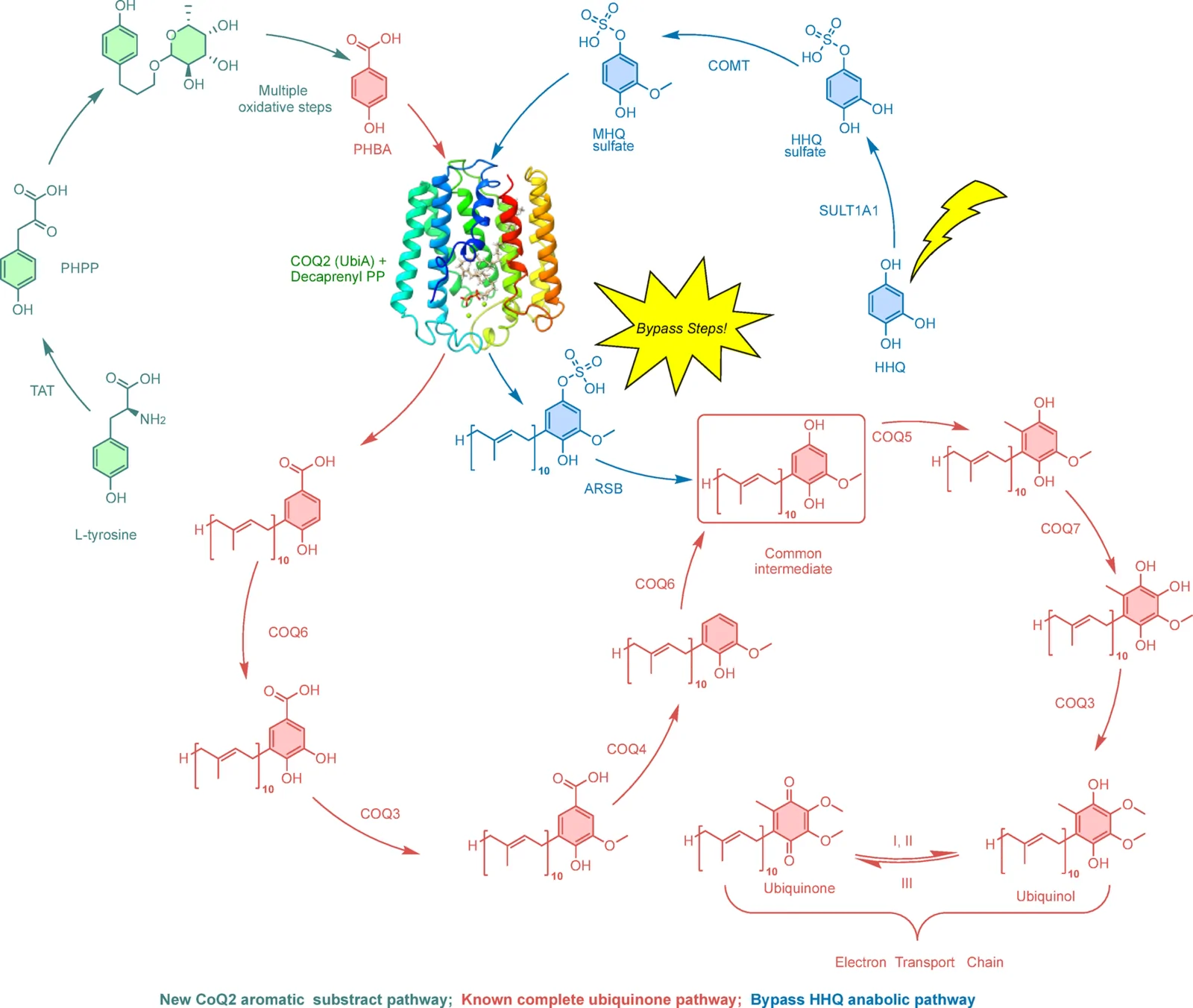
Schematic of known mitochondrial ubiquinol biosynthesis in mammals (red) that starts with PHBA as aromatic building block. A new pathway (blue, upper right) that begins with HHQ sharing some nonspecific COQ2 (UbiA, central) with PHBA on a key step of known model. HHQ bypass pathway, which avoids four key enzymatic reactions to obtain final ubiquinone product. A hypothetical PHBA cellular production (green, upper left) with *L*-tyrosine starts the pathway

In this study we investigated a new potential metabolic route for HHQ that integrates with the biosynthetic pathway of CoQ_10,_ revealing novel molecular interactions not been published until present. We performed molecular docking assays to evaluate the ability of HHQ and its likely derivative, methoxy-1,4-hydroquinone-sulfate (MHQ sulfate), to act as aromatic substrate of human COQ2. We believe that HHQ is most likely acting as an acceptor of prenyl pyrophosphate chains in initial steps of the biosynthesis of ubiquinol (Fig. 7).

The molecular docking model applied in the present study was based on known biochemical pathways published and are in accordance to the results presented in this paper [29, 42]. Literature has demonstrated evidence that UbiA may be affected by mutations in various active sites, playing an important role in subtract selectivity. One of the studies that supports our theory was performed in *Escherichia coli*, in which human homologous ApUbiA is expressed in a mutant *E. coli* containing inactive cofactor geranyl S-pyrophosphate. The results allowed for the determination of the enzyme’s active site, as the precursor PHBA can be crystallized in the unreacted form near the active site [29].

Also of great support is a recent study published by María de los Ángeles Vargas-Pérezare et al. (2024) [42], which evaluates strategies based on COQ2 homology that explores possible substrate binding sites of available ligand-bound homologs with solved structures. This study evaluates structural superposition in the AlphaFold predicted model for COQ2 and highlight some residues located on the central cavity or the matrix loops. From a series of different variants associated with primary CoQ deficiency onto the COQ2, a special attention is a mutation predicted in this study at the p.Arg123His site.

We have further performed a series of biochemical experiments on HepG2/IRM to support our theory by assessing specific in vitro pathways of CoQ_10_. We first explored the activity of ETC complex 1 (C1) after all treatments. The results in this study highlighted a substantial increase in C1 after HHQ exposition (+ 70% of control) to HepG2/IRM compared to the known aromatic precursor for this purpose PHBA (+ 10% of control), indicating a great activation of NADH: ubiquinone oxidoreductase. According to publications, PHBA administration improves CoQ_10_ levels in animal models with induced deficiency in tyrosine catabolism, validating the thesis of this carboxylic acid being the precursor of CoQ_10_ [28].

PABA was also tested and a significant reduction in Complex I activity (−42% of control) was observed in our study. PABA is described as a precursor to other forms of CoQ found in yeast and bacteria (CoQ6 and CoQ9) using a biochemical pathway similar to humans, with the intermediate 3-(*n*-prenyl)−4-aminobenzoate being active in CoQ biosynthesis [43]. PHBA administration improves CoQ_10_ levels in animal models with induced deficiency in tyrosine catabolism, validating the thesis of this carboxylic acid being the precursor of CoQ_10_^26^. PABA showed a significant reduction in Complex I activity, probably due to the inhibitory effect on the active site of the UBiA enzyme, probably due to the inhibitory effect on the active site of the UBiA enzyme, or even for producing an inactive prenylated compound. Apart from the suppressive activity found for PABA, Complex 1 activity revealed suppressive levels also for metformin (METF) treated group. METF is an antidiabetic drug that induces anaerobic glycolysis, thereby reducing oxidative phosphorylation in mitochondria, increasing lactic acid production, and consequently, reducing electron transport chain activity as a whole [44].

CoQ is reduced by NADH through Complex I activity [45]. C1 is the first fixed protein complex in ETC, with ubiquinone and cytochrome C as mobile components. Ubiquinone, also known as Coenzyme Q or CoQ_10,_ is a lipophilic component that moves through the lipid membrane on the inner face of the mitochondrial matrix, acting as an energy carrier from complexes I and II to complex III by interconversion of the ubiquinone: ubiquinol redox pair. In the ETC, CoQ is in a permanent equilibrium between a reduced form after receiving two electrons, CoQH2 or ubiquinol, and an oxidized form, CoQ or ubiquinone [46].

CoQ is reduced by mitochondrial glycerol 3-phosphate dehydrogenase, which receives electrons from sugars, but can also receive electrons from the electron transport flavoprotein dehydrogenases family, key enzymes of the fatty acid β-oxidation and branched-chain amino acid oxidation pathways [47, 48]. Furthermore, CoQ can be reduced by mitochondrial dihydroorotate dehydrogenase, a key enzyme of the pyrimidine nucleotide biosynthesis pathway [49], which can influence the reactive oxygen species production, partially contributing to ROS signaling in mitochondria [50]. There are many different points of ROS production in the respiratory chain, however the flavin mononucleotide (FMN) site, or the more controversial CoQ-binding site in Complex I, and the CoQ_10_ site of Complex III, are reported to be the most important ones [51, 52].

Recent results from our group have shown that ROS levels increased in cells with insulin resistant model (HepG2/IRM), but were maintained lower in the group treated with HHQ than those observed in the control group [22]. It is well reported that most of the reactive oxygen species production takes place in the mitochondrial respiratory chain [53], mainly in the form of superoxide that is transformed to hydrogen peroxide [54, 55]. Some reports highlight the importance of the retrograde electron transport (RET) mechanism that happens when accumulation of reduced CoQ occurs and generates superoxide, driving electrons throughout Complex I to NAD+, this mechanism balances the ubiquinone/ubiquinol ratio [56, 57].

ROS are generated inside the cells in the form of superoxide radical anion (O_2_^⋅−^) or hydrogen peroxide (H_2_O_2_). The respiratory chain is one of the major sites of metabolic ROS generation within the cell [58]. They are first generated in the form of O_2_^⋅−^, which are instable and very short-lived. O_2_^⋅−^ is rapidly converted to the more stable H_2_O_2_ by superoxide dismutase located in the cytosol and the mitochondrial inter-membrane space and matrix. Although production of superoxide anions (O_2_^⋅−^) are reported to be stimulated by HHQ [12], our results showed no increase in SOD activity after insulin and HHQ exposition, even though a generous increase in C1 was observed after HHQ treatment. It is important to remind that compounds like PABA, PHBA, and METF tested in the present study exhibited significant increases in SOD activity after insulin intake. SOD activity is directly linked to the mitochondrial ability to deplete ROS, especially the superoxide radical anion (O_2_^⋅−^).

Not only reactive oxygen species are involved as important signal compounds in many systems that control a host of cellular functions. Reactive nitrogen species are also key players and together with ROS represent small inorganic molecules that can diffuse easily to their site of action and disrupt redox cell state. Nitric oxide (⋅NO) has been reported to occur preferably in the cytosol and on the inner cell plasma membrane and its levels are related to metabolic changes not only directly linked to energy production but also to cellular signaling metabolism. A great suppressive power on ⋅NO production was observed in this study after HHQ exposition, with a reduction of 25% compared to the negative control in the presence of insulin. ⋅NO levels were also suppressed for PABA, PHBA and METF, although in a less expressive manner. It is known that nitric oxide can bind to many heme-containing prosthetic groups in enzymes and alter their function. This may lead to changes in electron transport chain activity in mitochondria. In some enzymes, ⋅NO is also able to bind to iron not associated to heme groups. An example are enzymes containing iron-sulphur complexes such as complex 1 of the mitochondria electron transport chain [59]. Those interactions are possible to happen and may sustain the suppressive levels on ⋅NO production after HHQ exposition.

Literature points that oxidative stress following HHQ consumption is likely to decreases fat catabolism by reducing ⋅NO availability. An indirect calorimetry study showed that one-week ingestion of HHQ-reduced coffee led to significantly higher fat utilization in healthy humans [1]. Further studies of the group revealed that the reduction of HHQ levels in roasted coffee stimulated fat utilization by increasing ⋅NO availability (Keiko Ishida, et al., (2018). One theory taken is that HHQ-derived superoxide anions can rapidly inactivate ⋅NO to form peroxynitrite and inhibit NOS activity [60]. There is an clear involvement of the endothelium-derived vasodilator NO in regulation of fatty acid metabolism [61–63]. In vascular tissues, nitric oxide activates guanylyl cyclase, which causes muscle relaxation and blood flow through the vassels. Reduction of the bioavailability of ⋅NO has been associated with vascular metabolism as an effect of HHQ intake in rodents [18, 19] and humans [14, 21].

Nitric oxide is also involved in many signaling processes of neuronal physiology. It has been implicated in the regulation of neurotransmitter release and might affect differentiation and regeneration of neuronal tissue. In an interesting manner, coenzyme Q_10_ is reported to be present in the Golgi complex of neuronal cells as a lipid soluble antioxidant synthesized in an endogenous way. It has been demonstrated that Q_10_ could help to balance redox signaling pathway counteracting the excess of ROS production that is responsible for cellular oxidative damage such as lipid peroxidation, protein nitrosylation and DNA oxidation [64]. It is known that Q_10_ is a mobile lipophilic electron carrier critical for electron transfer in the mitochondrial and Golgi compartments and plasma membrane. Among distinct functions, Q_10_ is also responsible for NAD(P)H-oxidoreductase-dependent reactions, which includes NO synthesis [65].

The results obtained using in vitro experiments presented solid evidence of increased ubiquinone production by human hepatocytes when treated with HHQ for 24 h, especially substantial increases in the activity of complexes I and II, stabilization of SOD activity, reduction in nitric oxide expression and increasing ubiquinol catabolism.

Molecular docking studies with MHQ-4-sulfate variant show that the sulfate groups are oriented towards the guanidinium-containing side chain of arginine p.123, similar to the carboxylate group of PHBA, stabilized at an average distance of 3 Å. The hydroxyl groups at C1 stabilize at an average distance of 2.6 Å from the O-C position of the substrate geranyl pyrophosphate, consistent with experiments conducted by Yang, et al., (2023) [29] using the same optimized UbiA model (PDB 4OD5). The molecular docking simulation with PHBA yielded nine stable binding poses, with binding energies ranging from − 5.7 to −5.2 kcal·mol⁻¹. Likely, MHQ-4-sulfate also produced nine binding poses, with binding energies ranging from − 6.2 to −4.0 kcal·mol⁻¹ (Fig. SI 21). Both substrates exhibited energetically favorable binding poses that are compatible with the completion of the enzymatic reaction.

Our simulations indicate that the MHQ-4-sulfate compound is the most viable derivative to be used as a substrate for COQ2. A study presenting results in accordance with Acosta, et al., (2019) [41], using cell cultures showed that vanillic acid is also assimilated by COQ2, leading to the methoxylated intermediate capable of supplementing deficiencies in COQ_10_ production caused by COQ3 mutations. This evidence reinforces the thesis that MHQ-4-sulfate is a viable substrate for COQ2, since the compound shares a similar chemical configuration to vanillate.

From the results here presented, which highlights the increase in the activities of ETC complexes I and II, the reduction in ⋅NO production, and the stabilization of SOD activity, authors are pointing to the assimilation of HHQ as a substrate in CoQ_10_ biosynthesis. HHQ showed superior results to PHBA in all experiments, with PHBA being the known aromatic precursor of CoQ_10_. Computational simulation assays demonstrated that sulfated derivatives of HHQ can be assimilated by native human COQ2. The hypothesis raised in this study requires the action of a mitochondrial sulfatase enzyme (SULF), such as aryl sulfatase B (ARSB) [66], and the Golgi complex for the hydrolysis of the sulfate ester and the consequent release of hydroquinone intermediates for subsequent enzymes. It is worth noting that HHQ is an endogenous compound already cited as a product of PHBA catabolism. These authors study the possibility of HHQ being produced by the catabolism of catecholamine neurotransmitters by gut microbiota, followed by assimilation in a pathway similar to *p*-cresol sulfate. We also investigated the hypothesis of HHQ being produced independently of aromatic amino acids, being a product of D-Chiro-Inositol (DCI) catabolism in the liver. The pharmacological potential of HHQ and sulfated derivatives as drugs or dietary supplements for a series of disorders such as treatment of type II diabetes, diseases resulting from metabolic syndrome, and syndromes resulting from mutations affecting the biosynthetic pathway of CoQ_10_.

## Materials and methods

### Chemical compounds

HHQ (A22 × 03233 - Alfa Chemistry, purity 98%), PABA (A9878 - Sigma-Aldrich), PHBA (H20059 - Sigma-Aldrich) were suspended in ultrapure water and tests were conducted at final concentration of 5.10^− 5^ mol.L^− 1^. METF (317240 - Sigma-Aldrich) was tested with 5.10^− 6^ mol.L^− 1^, and TBHP (458139 - Sigma-Aldrich).

### Cell culture and cytotoxic assay

The established human HepG2 hepatoma cell line was obtained from the American Type Culture Collection (MD, USA). HepG2 cells were grown in Dulbecco’s modified Eagle’s medium (DMEM) supplemented with 10% fetal bovine serum with humidified atmosphere, 5% CO_2_ and 37 °C. Cell viability of HepG2 against different treatment was measured using the MTT assay in 96-well plates at a density of 8.10^4^ cells.mL^− 1^. After 24 h, cells were treated with different concentrations of extracts and incubated for 24 h. Negative controls were treated with the same amounts of solution for 24 h. MTT dye was placed in serum-free medium and and incubated at 37 °C for 2 h in humidified 5% CO_2_ atmosphere. To obtain a formazan violet product, 100 mL of DMSO was dissolved in each well. Absorbance was measured using a microplate reader (Spectra Max M2e, Molecular Devices, USA) at l 570 nm. All readings were compared with the control, which represented 100% viability. Each experiment was performed in triplicate and independently repeated at least four times.

### Insulin resistance model (HepG2/IRM)

The experiments in this study followed similar protocol published recently by our group for insulin resistance model [22] and was adapted from Kheirollahzadeh et al. (2022) [67]. Briefly, to establish an insulin-resistant cell model (HepG2/IRM), a gradient of insulin at different concentrations was initially tested for viability through MTT. Cells were seeded in 96-well plates at 8.10^4^ cells.mL^− 1^ in DMEM supplemented with a high glucose concentration (2.5.10^− 2^ mol.L^− 1^). After 24 h incubation, cells were treated with insulin at concentrations of 10^− 4^ mol.L^− 1^ to 10^− 7^ mol.L^− 1^. Best parameters for insulin concentration was selected for IR model and further experiments were processed with insulin at 5.10^− 6^ mol.L^− 1^ for 24 h. Further treatments were performed with different compounds and left for 24 h, as following: HHQ 5.10^− 5^ mol.L^− 1^; PABA 5.10^− 5^ mol.L^− 1^; PHBA 5.10^− 5^ mol.L^− 1^; METF (5.10^− 6^ mol.L^− 1^) and TBHP (2.10^− 5^ mol.L^− 1^).

### Mitochondrial complex I (CI) and complex II (CII) activities

The methodology of Spinazzi et al. (2012) [45] was used to verify the activity of complex I (NADH: ubiquinone oxidoreductase) of the mitochondrial electron transport chain. Complex I activity was measured at λ 600 nm for 3 min with positive control rotenone (10^− 3^mol.L^− 1^). Enzymatic activity was read at 37 °C using a spectrophotometer (Shimadzu model UV-1700). The results were presented as percentages of control. Each value comes from four readings for each sample (2 with and 2 without inhibitor). The assessment of Mitochondrial Complex II (CII, succinate dehydrogenase) activity was also adapted by [45] and used to verify the activity of complex II of the mitochondrial electron transport chain. The cell lysate was exposed to 2-thenoyltrifluoroacetone TTFA 5.10^− 4^ mol.L^− 1^, and the activity was monitored for 3 min at λ 600 nm (spectrophotometer Shimadzu model UV-1700) at 37 °C, after an incubation period of 10 min. The results were presented as percentage in relation to the control.

### Nitric oxide (⋅NO) assay

Cells were plated in 6-well plates at 3.10^5^ cells mL^− 1^ and exposed to different treatments, similar protocol applied to the ATP assay. Cells were lysed after treatment using 100 µL of RIPA buffer (5.10^− 2^ mol.L^− 1^ Tris - pH 8,0; 1.5.10^− 1^mol.L^− 1^ NaCl; 1,0% NP-40; 0,5% sodium deoxycholate; 0,1% SDS) per well, during 10 min at 4 °C. Cell lysate was collected from each well and centrifuged for 10 min at 14.000 rpm. Supernatant was collected and total protein content was measured using a Qubit protein assay kit (Invitrogen, Thermo Fisher Scientific), according to manufactory instructions. Levels of nitric oxide were determined as nitrite (NO_2_^−^) production, which is a NO metabolite, using the Griess reaction-based method [68]. For the assay, 50 µL of the sample were reacted with an equal volume of Griess reagent (0.1% naphthyl ethylenediamine and 1% sulfanilamide in 5% H_3_PO_4_) for 10 min at room temperature, and the absorbance read at λ 550 nm in a microplate reader. Sodium nitroprusside was used as the standard. The results were expressed as percentage of control.

### Superoxide dismutase (SOD) activity

Cells lysates were collected after treatment with RIPA buffer for each well using a 6-well plate, using a similar protocol as applied in NO assay. SOD activity was measured by the inhibition of self-catalytic adrenochrome formation rate at λ 480 nm, in a reaction medium containing 1 mmol/L adrenaline (pH 2.0) and 5.10^− 2^ mol.L^− 1^ glycine (pH 10.2) at 30 °C for 3 min [69]. The results from kinetics were presented as percentages.

### Adenosine triphosphate (ATP) activity

ATP activity was monitored through the Luminescent ATP Determination Kit (A22066 -Sigma Aldrich). Quantitative determination of ATP bioluminescence assay is evaluated with recombinant firefly luciferase and its substrate D-luciferin. The assay is based on luciferaseʼs requirement for ATP in producing light. A standard curve was prepared with ATP concentrations ranging from 10^− 6^ mol.L^− 1^ to 10^− 5^ mol.L^− 1^, substituting ATP-containing samples for the ATP standard solutions provided in the kit with total equal volume of the experimental sample assays. Cells were plated in 6-well plates at 3.10^5^ cells.mL^− 1^ and exposed to different treatments. After adherence, cells were treated with the highest non-cytotoxic concentration of HHQ, precursor acids and controls for 24 h. Cell cultures were lysed using 100 uL of TE buffer solution (10^− 1^ mol.L^− 1^ Tris-base; 4.10^− 1^ mol.L^− 1^ EDTA; pH 7,5) and samples were heated for 7 min in water bath. Briefly, 10 uL of cell lysed was mixed with 90 uL of standard reaction solution provided in the kit. Measurement was performed using a Luminometer (SpectraMax L – Molecular Devices) with emission maximum ~ λ 560 nm at pH 7.8. Results are expressed in relative light unit (RLU) that measures the light emitted when ATP interacts with the luciferase enzyme, producing a bioluminescent reaction. RLU are the units used for most luminescence measurements and comparison between samples was taken using a ATP standard curve.

### HPLC-HRMS analysis

Prior to identification, cells were lysed with 150 uL of RIPA buffer (5.10–2 mol.L-1 Tris - pH 8,0; 1.5.10–1 mol.L-1 NaCl; 1,0% NP-40; 0,5% sodium deoxycholate; 0,1% SDS) per well, during 10 min at 4 °C. Equal amounts (75uL) of KCl 150mM and methanol were added and samples were vortexed and centrifuged for 5 min at 15.000 rpm. The supernatant was transferred to new tubes, and 250 µL of hexane was added. The samples were mixed again and centrifuged to extract the lipophilic fraction, then slowly dried under nitrogen gas. Matabolomic study was performed according Nicoll, et al., 2024 [70]. Analytical serie was carried out on UHPLC system (Shimadzu Proeminence 20 A series) coupled to electrospray ionization q-TOF high resolution mass spectrometry (ESI–qTOF-HRMS) to detect negative and positive ions. Chromatographic system configuration: Collumn UCT Selectra^®^ C18 100 × 2.1 mm, particle 3 μm performed at 25 °C. Binary gradient components: water: formic acid 0.1% (A) and Acetonitrile: formic acid 0.1% (B); gradient program: 0.0–0.1 min (98% A: 2%B), 2.0–32.0 min (44% A: 66% B), 32.0–35.0 min (98% A: 2%B); flow 0.2 mL min^− 1^; injection volume 20 µL. ESI–qTOF-HRMS setup: MicroTOF Q-II series spectrometer (Bruker Daltonics), ESI parameters: Nebulizer pressure (N_2_): 2.0 bar, dry heater: 200 °C, dry gas flow: 6.0 L min^− 1^, capillary set: 2500 V, MS parameters: set scan range: 80–2000 m/z, Funnel 1 RF: 300 Vpp, set collision cell (broad band Collision Induced Dissociation – bbCID): 25–50 eV, Acquisition data mode: MRM to MS and bbCID to MS2. Data extraction and treatment: Bruker Compass DataAnalysis software, version 4.3 (Build 110.102.1532); data filters used: Base Peak Chromatogram (BPC) of all MS, bbCID-BPC of all MS2, Extracted Ion Chromatogram (EIC) of all MS to integration areas.

### Molecular modeling studies

Computational experiments were performed on UCSF Chimera version 1.17.3 [71] with AutoDock Vina plug-in version 1.1.2 [72]. Docking images were generated on ChimeraX version 1.7.1 [73]. The human COQ2 isoform 1 (*h*COQ2) [26] were generated from Phyre2 server [74] using ApUbiA homolog of *Aeropyrum pernix* K1 (PDB 4OD5) as fold [75]. These PDB model 4OD5 is the available reference containing both n-prenyl-pyrophosphate and PHBA substrates crystallized at the lowest energy coordinates of the enzyme’s site, besides maintaining a high homology degree (63%) of *h*COQ2. The aromatic ligands were modeled in their anionic forms with a pontual charge on the sulfate group. The minimized energy conformers were generated using the ChemOffice 21.0.0.28 package (free trial).

### Statistical analysis

The results were expressed as mean ± standard deviation (SD) of three independent experiments performed in triplicate, used to assess normal distribution data. One-way analysis of variance (ANOVA) was applied, followed by Tukey post-hoc test. Statistically significant of mean differences was accepted at a level of p *≤* 0.05. Statistical analyses and plots were performed using OriginPro, Version 2024, OriginLab Corporation, Northampton, MA, USA.

## Supplementary Information

Below is the link to the electronic supplementary material.

Supplementary Material 1

## Data Availability

No datasets were generated or analysed during the current study.

## References

[1] Soga S, Ota N, Shimotoyodome A (2017) Reduction in hydroxyhydroquinone from coffee increases postprandial fat utilization in healthy humans: a randomized double-blind, cross-over trial. Biosci Biotechnol Biochem 81:1433–1435. 10.1080/09168451.2017.1301802 28322128

[2] Turunen M, Olsson J, Dallner G (2004) Metabolism and function of coenzyme Q. Biochim Biophys Acta - Biomembr 1660:171–199. 10.1016/j.bbamem.2003.11.012 14757233

[3] Hernández-Camacho JD, García-Corzo L, Fernández-Ayala DJM et al (2021) Coenzyme q at the hinge of health and metabolic diseases. Antioxidants. 10.3390/antiox10111785 PMC8615162 34829656

[4] Villalba JM, Navas P (2021) Regulation of coenzyme Q biosynthesis pathway in eukaryotes. Free Radic Biol Med 165:312–323. 10.1016/j.freeradbiomed.2021.01.055 33549646

[5] Alcázar-Fabra M, Rodríguez-Sánchez F, Trevisson E, Brea-Calvo G (2021) Primary coenzyme Q deficiencies: a literature review and online platform of clinical features to uncover genotype-phenotype correlations. Free Radic Biol Med 167:141–180. 10.1016/j.freeradbiomed.2021.02.046 33677064

[6] López-Lluch G (2021) Coenzyme Q homeostasis in aging: response to non-genetic interventions. Free Radic Biol Med 164:285–302. 10.1016/j.freeradbiomed.2021.01.024 33454314

[7] Gueguen N, Baris O, Lenaers G et al (2021) Secondary coenzyme Q deficiency in neurological disorders. Free Radic Biol Med 165:203–218. 10.1016/j.freeradbiomed.2021.01.017 33450382

[8] Schijvens AM, van de Kar NC, Bootsma-Robroeks CM et al (2020) Mitochondrial disease and the kidney with a special focus on CoQ10 deficiency. Kidney Int Rep 5:2146–2159. 10.1016/j.ekir.2020.09.044 PMC7710892 33305107

[9] Wang Y, Hekimi S (2022) The efficacy of coenzyme Q 10 treatment in alleviating the symptoms of primary coenzyme Q 10 deficiency: a systematic review. J Cell Mol Med 26:4635–4644. 10.1111/jcmm.17488 PMC9443948 35985679

[10] González-García P, Jiménez-Sánchez L, Corral-Sarasa J et al (2025) The treatment of primary CoQ de fi ciency requires the targeting of multiple pathogenic mechanisms. Commun Med 5:1–13. 10.1038/s43856-025-01000-8 PMC12246469 40640372

[11] Steenberge LH, Rogers S, Sung AY et al (2024) Coenzyme Q 4 is a functional substitute for coenzyme Q 10 and can be targeted to the mitochondria. J Biol Chem 300:107269. 10.1016/j.jbc.2024.107269 PMC11087978 38588811

[12] Ishida K, Misawa K, Yamamoto M, Shimotoyodome A (2018) Hydroxyhydroquinone impairs fat utilization in mice by reducing nitric oxide availability. J Physiol Sci 68:855–864. 10.1007/s12576-018-0605-9 PMC6223984 29572772

[13] Ochiai R, Jokura H, Suzuki A et al (2004) Green coffee bean extract improves human vasoreactivity. Hypertens Res 27:731–737. 10.1291/hypres.27.731 15785008

[14] Ochiai R, Chikama A, Kataoka K et al (2009) Effects of hydroxyhydroquinone-reduced coffee on vasoreactivity and blood pressure. Hypertens Res 32:969–974. 10.1038/hr.2009.132 19713967

[15] Angerer J, Bernauer U, Chambers C et al (2012) Opinion on 1,2,4-trihydroxybenzene (COLIPA n^o^ A33) (Report SCCS/1452/11)

[16] Wada H, Oyama K, Kamae R et al (2019) Zinc-dependent and independent actions of hydroxyhydroquinone on rat thymic lymphocytes. Drug Chem Toxicol 42:496–501. 10.1080/01480545.2018.1429462 29421941

[17] Liu Y, Caruso J, Zhang H (2021) Developing an effective approach for microbial biosynthesis of hydroxyhydroquinone. Biochem Eng J 168:107929. 10.1016/j.bej.2021.107929

[18] Suzuki A, Fujii A, Yamamoto N et al (2006) Improvement of hypertension and vascular dysfunction by hydroxyhydroquinone-free coffee in a genetic model of hypertension. FEBS Lett 580:2317–2322. 10.1016/j.febslet.2006.03.047 16579992

[19] Suzuki A, Fujii A, Jokura H et al (2008) Hydroxyhydroquinone interferes with the chlorogenic acid-induced restoration of endothelial function in spontaneously hypertensive rats. Am J Hypertens 21:23–27. 10.1038/ajh.2007.3 18091740

[20] Ochiai R, Nagao T, Katsuragi Y et al (2008) Effects of hydroxyhydroquinone-reduced coffee in patients with essential hypertension. J Heal Sci 54:302–309. 10.1248/jhs.54.302

[21] Yamaguchi T, Chikama A, Mori K et al (2008) Hydroxyhydroquinone-free coffee: a double-blind, randomized controlled dose-response study of blood pressure. Nutr Metab Cardiovasc Dis 18:408–414. 10.1016/j.numecd.2007.03.004 17951035

[22] Santos PR, Danetti S, Rastegar AJ, de Souza WV, Frassini R, Scariot FJ, Moura S, Roesch-Ely M (2024) Hydroxyhydroquinone and quassinoids as promising compounds with hypoglycemic activity through redox balance. Compounds 4:17–36. 10.3390/compounds4010002

[23] Sanz A, Navas P (2018) Editorial: coenzyme Q redox state and cellular homeostasis. Front Physiol 9:1–2. 10.3389/fphys.2018.00912 PMC6077213 30104979

[24] Scialo F, Sanz A (2021) Coenzyme Q redox, signalling and longevity. Free Radic Biol Med 164:187–205. 10.1016/j.freeradbiomed.2021.01.018 33450379

[25] Nicoll CR, Alvigini L, Gottinger A et al (2024) In vitro construction of the COQ metabolon unveils the molecular determinants of coenzyme Q biosynthesis. Nat Catal 7:148–160. 10.1038/s41929-023-01087-z PMC7615680 38425362

[26] Forsgren M, Attersand A, Lake S et al (2004) Isolation and functional expression of human COQ2, a gene encoding a polyprenyl transferase involved in the synthesis of CoQ. Biochem J 382:519–526. 10.1042/BJ20040261 PMC1133808 15153069

[27] Nazlı Gönç E, Çakır M, Düzova A et al (2015) A rare case of neonatal diabetes due to COQ2 gene mutation. JCRPE J Clin Res Pediatr Endocrinol 7:16

[28] Herebian D, Seibt A, Smits SHJ et al (2017) 4-Hydroxybenzoic acid restores CoQ10 biosynthesis in human COQ2 deficiency. Ann Clin Transl Neurol 4:902–908. 10.1002/acn3.486 PMC5740244 29296619

[29] Yang E, Yao Y, Liu Y et al (2023) A Gatekeeper Residue Controls Aromatic Acceptor Specificity of the PHB-Type UbiA Prenyltransferases. ACS Catal 13:13717–13728. 10.1021/acscatal.3c04085

[30] Rutherford K, Le Trong I, Stenkamp RE, Parson WW (2008) Crystal structures of human 108V and 108M catechol O-methyltransferase. J Mol Biol 380:120–130. 10.1016/j.jmb.2008.04.040 18486144

[31] D’Agostino GD, Chaudhari SN, Devlin AS (2024) Host–microbiome orchestration of the sulfated metabolome. Nat Chem Biol 20:410–421. 10.1038/s41589-023-01526-9 PMC11095384 38347214

[32] Chan TS, O’Brien PJ (2003) Hepatocyte metabolism of coenzyme Q1 (ubiquinone-5) to its sulfate conjugate decreases its antioxidant activity. BioFactors 18:207–218. 10.1002/biof.5520180223 14695936

[33] Denizot F, Lang R (1986) Rapid colorimetric assay for cell growth and survival. Modifications to the tetrazolium dye procedure giving improved sensitivity and reliability. J Immunol Methods 89:271–277 10.1016/0022-1759(86)90368-6 3486233

[34] DeBarber AE, Bleyle LA, Roullet JBO, Koop DR (2004) ω-Hydroxylation of farnesol by mammalian cytochromes P450. Biochim Biophys Acta - Mol Cell Biol Lipids 1682:18–27. 10.1016/j.bbalip.2004.01.003 15158752

[35] Johnston JB, Ouellet H, Podust LM, de Montellano PR (2011) Structural control of cytochrome P450-catalyzed ω- hydroxylation. Arch Biochem Biophys 507:86–94. 10.1016/j.abb.2010.08.011.Structural PMC2997901 20727847

[36] Nakamura T, Ohno T, Hamamura K, Sato T (1999) Metabolism of coenzyme Q10: Biliary and urinary excretion study in guinea pigs. BioFactors 9:111–119. 10.1002/biof.5520090205 10416022

[37] Bentinger M, Dallner G, Chojnacki T, Swiezewska E (2003) Distribution and breakdown of labeled coenzyme Q10 in rat. Free Radic Biol Med 34:563–575. 10.1016/S0891-5849(02)01357-6 12614845

[38] Zhou Y, Fukuda T, Hang Q et al (2017) Inhibition of fucosylation by 2-fluorofucose suppresses human liver cancer HepG2 cell proliferation and migration as well as tumor formation. Sci Rep 7:1–12. 10.1038/s41598-017-11911-9 PMC5599613 28912543

[39] Sosicka P, Ng BG, Pepi LE et al (2022) Origin of cytoplasmic GDP-fucose determines its contribution to glycosylation reactions. J Cell Biol. 10.1083/jcb.202205038 PMC9441714 36053214

[40] Schwarz M (2004) Pathways and defects of bile acid synthesis: Insights from in vitro and in vivo experimental models. Drug Discov Today Dis Model 1:205–212. 10.1016/j.ddmod.2004.10.004

[41] Acosta Lopez MJ, Trevisson E, Canton M et al (2019) Vanillic acid restores coenzyme Q biosynthesis and ATP production in human cells lacking COQ6. Oxid Med Cell Longev. 10.1155/2019/3904905 PMC6652073 31379988

[42] Vargas-Pérez M, Devos DP, López-Lluch G (2024) An AlphaFold structure analysis of COQ2 as key a component of the coenzyme Q synthesis complex. Antioxidants (Basel) 13:1–17. 10.3390/antiox13040496 PMC11047366 38671943

[43] Pierrel F, Hamelin O, Douki T et al (2010) Involvement of mitochondrial ferredoxin and para-aminobenzoic acid in yeast coenzyme q biosynthesis. Chem Biol 17:449–459. 10.1016/j.chembiol.2010.03.014 20534343

[44] Defronzo R, Fleming GA, Chen K, Bicsak TA (2016) Metformin-associated lactic acidosis: current perspectives on causes and risk. Metabolism 65:20–29. 10.1016/j.metabol.2015.10.014 26773926

[45] Spinazzi M, Casarin A, Pertegato V et al (2012) Assessment of mitochondrial respiratory chain enzymatic activities on tissues and cultured cells. Nat Protoc 7:1235–1246. 10.1038/nprot.2012.058 22653162

[46] Alcázar-Fabra M, Navas P, Brea-Calvo G (2016) Coenzyme Q biosynthesis and its role in the respiratory chain structure. Biochim Biophys Acta - Bioenerg 1857:1073–1078. 10.1016/j.bbabio.2016.03.010 26970214

[47] Bentinger M, Tekle M, Dallner G (2010) Coenzyme Q - biosynthesis and functions. Biochem Biophys Res Commun 396:74–79. 10.1016/j.bbrc.2010.02.147 20494114

[48] Watmough NJ, Frerman FE (2010) The electron transfer flavoprotein: Ubiquinone oxidoreductases. Biochim Biophys Acta - Bioenerg 1797:1910–1916. 10.1016/j.bbabio.2010.10.007 20937244

[49] Evans DR, Guy HI (2004) Mammalian pyrimidine biosynthesis: fresh insights into an ancient pathway. J Biol Chem 279:33035–33038. 10.1074/jbc.R400007200 15096496

[50] Hey-Mogensen M, Goncalves RLS, Orr AL, Brand MD (2014) Production of superoxide/H2O2 by dihydroorotate dehydrogenase in rat skeletal muscle mitochondria. Free Radic Biol Med 72:149–155. 10.1016/j.freeradbiomed.2014.04.007 24746616

[51] Quinlan CL, Perevoshchikova IV, Hey-Mogensen M et al (2013) Sites of reactive oxygen species generation by mitochondria oxidizing different substrates. Redox Biol 1:304–312. 10.1016/j.redox.2013.04.005 PMC3757699 24024165

[52] Hirst J (2013) Mitochondrial complex i. Annu Rev Biochem 82:551–575. 10.1146/annurev-biochem-070511-103700 23527692

[53] Murphy MP (2009) How mitochondria produce reactive oxygen species. Biochem J 417:1–13. 10.1042/BJ20081386 PMC2605959 19061483

[54] Borgstahl GE, Parge HE, Hickey MJ et al (1992) The structure of human mitochondrial manganese superoxide dismutase reveals a novel tetrameric interface of two 4-helix bundles. Cell 71:107–118. 10.1016/0092-8674(92)90270-m 1394426

[55] Han D, Canali R, Rettori D, Kaplowitz N (2003) Effect of glutathione depletion on sites and topology of superoxide and hydrogen peroxide production in mitochondria. Mol Pharmacol 64:1136–1144. 10.1124/mol.64.5.1136 14573763

[56] Hirst J, King MS, Pryde KR (2008) The production of reactive oxygen species by complex I. Biochem Soc Trans 36:976–980. 10.1042/BST0360976 18793173

[57] Pryde KR, Hirst J (2011) Superoxide is produced by the reduced flavin in mitochondrial complex I: a single, unified mechanism that applies during both forward and reverse electron transfer. J Biol Chem 286:18056–18065. 10.1074/jbc.M110.186841 PMC3093879 21393237

[58] van Soest DMK, Polderman PE, den Toom WTF et al (2024) Mitochondrial H_2_O_2_ release does not directly cause damage to chromosomal DNA. Nat Commun. 10.1038/s41467-024-47008-x PMC10978998 38548751

[59] Sharma LK, Lu J, Bai Y (2009) Mitochondrial respiratory complex I: structure, function and implication in human diseases lokendra. Curr Med Chem 16:1266–1277. 10.4049/jimmunol.1801473.The PMC4706149 19355884

[60] Crabtree MJ, Channon KM (2011) Synthesis and recycling of tetrahydrobiopterin in endothelial function and vascular disease. Nitric Oxide - Biol Chem 25:81–88. 10.1016/j.niox.2011.04.004 PMC5357050 21550412

[61] García-Villafranca J, Guillén A, Castro J (2003) Involvement of nitric oxide/cyclic GMP signaling pathway in the regulation of fatty acid metabolism in rat hepatocytes. Biochem Pharmacol 65:807–812. 10.1016/S0006-2952(02)01623-4 12632570

[62] Le Gouill E, Jimenez M, Binnert C et al (2007) Endothelial nitric oxide synthase (eNOS) knockout mice have defective mitochondrial β-oxidation. Diabetes 56:2690–2696. 10.2337/db06-1228 17682093

[63] Jobgen WS, Fried SK, Fu WJ et al (2006) Regulatory role for the arginine-nitric oxide pathway in metabolism of energy substrates. J Nutr Biochem 17:571–588. 10.1016/j.jnutbio.2005.12.001 16524713

[64] Mugoni V, Postel R, Catanzaro V et al (2013) Ubiad1 is an antioxidant enzyme that regulates eNOS activity by CoQ10 synthesis. Cell 152:504–518. 10.1016/j.cell.2013.01.013 PMC3574195 23374346

[65] Navas P, Villalba JM, de Cabo R (2007) The importance of plasma membrane coenzyme Q in aging and stress responses. Mitochondrion 7:34–40. 10.1016/j.mito.2007.02.010 17482527

[66] Bhattacharyya S, Feferman L, Tobacman JK (2016) Restriction of aerobic metabolism by acquired or innate Arylsulfatase B deficiency: a new approach to the Warburg effect. Sci Rep. 10.1038/srep32885 PMC5015117 27605497

[67] Kheirollahzadeh F, Eftekhari E, Ghollasi M, Behzadi P (2022) Anti-hyperglycemic effects of Eryngium billardierei F. Delaroche extract on insulin-resistance HepG2 cells in vitro. Mol Biol Rep 49:3401–3411. 10.1007/s11033-022-07171-0 35092562

[68] Green LC, Tannenbaum SR, Goldman P (1981) Nitrate Synthesis in the Germfree and Conventional Rat. Sci (80-) 212:56–58. 10.1126/science.6451927 6451927

[69] Bannister JV, Calabrese L (1987) Assays for superoxide dismutase. Methods Biochem Anal. 10.1002/9780470110539.ch5 3033431

[70] Nicoll CR, Alvigini L, Gottinger A et al (2024) In vitro construction of the COQ metabolon unveils the molecular determinants of coenzyme Q biosynthesis. Nat Catal. 10.1038/s41929-023-01087-z PMC7615680 38425362

[71] Pettersen EF, Goddard TD, Huang CC et al (2004) UCSF Chimera - A visualization system for exploratory research and analysis. J Comput Chem 25:1605–1612. 10.1002/jcc.20084 15264254

[72] Trott O, Olson AJ (2010) AutoDock Vina: Improving the speed and accuracy of docking with a new scoring function, efficient optimization, and multithreading. J Comput Chem 31:455–461. 10.1002/jcc.21334 PMC3041641 19499576

[73] Meng EC, Goddard TD, Pettersen EF et al (2023) UCSF ChimeraX: Tools for structure building and analysis. Protein Sci 32:1–13. 10.1002/pro.4792 PMC10588335 37774136

[74] Kelley LA, Mezulis S, Yates CM et al (2015) The Phyre2 web portal for protein modeling, prediction and analysis. Nat Protoc 10:845–858. 10.1038/nprot.2015-053 PMC5298202 25950237

[75] Cheng W, Li W (2014) Structural insights into ubiquinone biosynthesis in membranes. Science 343:878–881. 10.1126/science.1246774 PMC4390396 24558159

