# Supplementary material for: Hydroxyhydroquinone as a novel modulator of mitochondrial metabolism with potential to restore CoQ10 homeostasis
Source: Mol Cell Biochem. 2026 Aug 7;481(9):4611–28. doi: 10.1007/s11010-026-05687-8 (PMC13638691; doi:10.1007/s11010-026-05687-8)
Supplement: Supplementary file 1 [file 11010_2026_5687_MOESM1_ESM.docx]

**Molecular and Cellular Biochemistry**

Supplementary Informations

Hydroxyhydroquinone as a novel modulator of mitochondrial metabolism with potential to restore CoQ10 homeostasis

Paulo Roberto dos Santos^a^, Joseph Rastegar^b^, Mariana Roesch-Ely^b^*

^a^ Independent researcher.

^b^ Institute of Biotechnology, University of Caxias do Sul, Caxias do Sul 95070-560, Brazil.

**Fig. SI 1** LC-HRMS data treatment

Overlay of full Base Peak Chromatograms (BPC) (0-36 min) of 6 cell treatments obtained at negative ions polarity. Large peaks at 9.5, 18.0 and 28.0 min are background components of blank run performed with pure methanol (**A**). Highlighted region of all identified compounds (2-6 min) (**B**). Overlay of Extracted Ion Chromatograms (EIC) of 14 Identified metabolites of PABA treatment to negative ions polarity (**C**).

**Fig. SI 2** LC-HRMS data treatment

Overlay of full Base Peak Chromatograms (BPC) (0-36 min) of CTRL treatment obtained at positive ions polarity (**A**). Highlighted region of all identified compounds (2-6 min) (**B**). Overlay of Extracted Ion Chromatograms (EIC) of 3 identified metabolites of CTRL treatment to positive ions polarity (**C**).

**Fig. SI 3** HRMS-MS data analysis of Compound 1 (tyrosine catabolite) at 3.0 min

MS data of negative ions polarity (**A**) show two molecular adducts [M+formate-H]^-^, [M+Cl]^-^ and deprotonated molecule [M-H]^-^ at m/z 343.1395, 333.1110 and 297.1339. Fragmentation spectrum (**B**) shows the fucopyranose moiety at m/z 163.0609 and 3-(4-hydroxyphenyl) propanal at m/z 149.0608. The positive ions spectrum (**C**) shows protonated molecule adduct [M+H]^+^ at m/z 299.1503, protonated fucopyranose moiety at m/z 165.0765 and protonated 4-allylphenol at m/z 135.0811. Positive fragmentation spectrum (**D**) shows the dimetyliminium derivative of 3-(4-hydroxyphenyl) propanal at m/z 178.1232, *p*-quinone methide form of 4-allylphenol at m/z 133.0648 and cationic form of ethylbenzene at m/z 105.0704.

**Fig. SI 4** HRMS-MS data analysis of Compound A (CoQ10 catabolite) at 3.3 min

MS data of negative ions polarity (**A**) shows deprotonated molecule [M-H]^-^ at m/z 421.1868. Fragmentation spectrum (**B**) shows 3 *p*-quinone fragments at m/z 218.0934, 193.0501 and 121.0293. Tail fragment was detected at m/z 171.1027.

**Fig. SI 5** HRMS-MS data analysis of Compound B (CoQ10 catabolite) at 3.6 min

MS data of negative ions polarity (**A**) shows deprotonated molecule [M-H]^-^ at m/z 463.1972. Fragmentation spectrum (**B**) shows *p*-quinol fragment at m/z 221.1191. Two tail fragments was detected at m/z 169.0871 and 187.0974 (hydrated form).

**Fig. SI 6** HRMS-MS data analysis of Compound C (CoQ10 catabolite) at 3.7 min

MS data at 3.7 min at negative ions polarity (**A**) shows deprotonated molecule [M-H]^-^ at m/z 477.2131. Fragmentation spectrum (**B**) shows *p*-quinol fragment at m/z 221.1191. Tail fragment was detected at m/z 169.0870.

**Fig. SI 7** HRMS-MS data analysis of Compound 2 (Desmethoxy-CoQ1) at 3.8 min

MS data at 3.8 min to negative ions polarity (**A**) shows deprotonated molecule [M-H]^-^ at m/z 221.1183. Fragmentation spectrum (**B**) shows molecular deprotonated sodium derivative [M-2H+Na]^-^ at m/z 243.1002 and bimolecular monosodium cluster derivative [2M-2H+Na]^-^ at m/z 425.2269.

**Fig. SI 8** HRMS-MS data analysis of Compound D (CoQ10 catabolite) at 3.8 min

MS data at 3.8 min at negative ions polarity (**A**) shows deprotonated molecule [M-H]^-^ at m/z 403.1770. Fragmentation spectrum (**B**) shows *p*-quinol fragment at m/z 221.1181.

**Fig SI 9** HRMS-MS data analysis of Compound E (CoQ10 catabolite) at 4.0 min

MS data at 4.0 min at negative ions polarity (**A**) shows deprotonated molecule [M-H]^-^ at m/z 505.2085. Fragmentation spectrum (**B**) shows two *p*-quinol fragments at m/z 249.1133 and 221.1183, *p*-quinone fragment at m/z 218.0948 and tail fragment at m/z 171.2029.

**Fig. SI 10** HRMS-MS data analysis of Compound F (CoQ10 catabolite) at 4.2 min

MS data at 4.2 min at negative ions polarity (**A**) shows molecular formate adduct [M+formate-H]^-^ at m/z 459.2029. No attributed fragment was detected on fragmentation spectrum (**B**).

**Fig. SI 11** HRMS-MS data analysis of Compound G (CoQ10 catabolite) at 4.3 min

MS data at 4.3 min at negative ions polarity (**A**) shows deprotonated molecule [M-H]^-^ at m/z 519.2249. Fragmentation spectrum (**B**) shows *p*-quinol fragment at m/z 221.1190 and tail fragment at m/z 171.1025.

**Fig. SI 12** HRMS-MS data analysis of Compound H (CoQ10 catabolite) at 4.5 min

MS data at 4.5 min at negative ions polarity (**A**) shows molecular formate adduct [M+formate-H]^-^ at m/z 459.2033 and bimolecular monoformate cluster derivative [2M-2H+formate]^-^ at m/z 873.4076. No attributed fragment was detected on fragmentation spectrum (**B**). The positive ions spectra (**C,D**) show 5 molecular adducts including protonated molecule [M+H]^+^ at m/z 415.2128, sodium adduct [M+Na]^+^ at m/z 437.1951, potassium adduct [M+K]^+^ at m/z 453.1692, ammonium adduct [M+NH_3_]^+^ at m/z 432.2381 and alkenyl cationic cluster [M+C_9_H_9_]^+^ at m/z 531.2755. Main fragment detected in positive polarity: Molecular acylium form [M-OH]^+^ at m/z 397.2018, *p*-quinone fragments at m/z 281.1386 and 263.1278, tail fragments with acylium form at m/z 135.0804 and 133.0648, 1,4-dimethyl tropylium ion at m/z 119.0859.

**Fig. SI 13** HRMS-MS data analysis of Compound I (CoQ10 catabolite) at 4.6 min

MS data at 4.6 min at negative ions polarity (**A**) shows deprotonated molecule [M-H]^-^ at m/z 445.1874. Fragmentation spectrum (**B**) shows *p*-quinol fragment at m/z 221.1183 and tail fragment at m/z 171.1025.

**Fig. SI 14** HRMS-MS data analysis of Compound J (CoQ10 catabolite) at 4.7 min

MS data at 4.7 min of negative ions polarity (**A**) shows molecular formate adduct [M+formate-H]^-^ at m/z 459.2029. No attributed fragment was detected on fragmentation spectrum (**B**).

**Fig. SI 15** HRMS-MS data analysis of Compound K (CoQ10 catabolite) at 4.7 min

MS data at 4.7 min at negative ions polarity (**A**) shows deprotonated molecule [M-H]^-^ at m/z 533.2398. Fragmentation spectrum (**B**) shows tail fragment at m/z 171.1024.

**Fig. SI 16** HRMS-MS data analysis of Compound 3 (deoxycholic acid) at 4.9 min

MS data at 4.9 min of negative ions polarity spectra (**A,B**) show deprotonated molecule [M-H]^-^ at m/z 391.2863, molecular formate adduct [M+formate-H]^-^ at m/z 437.2922, dimmer [2M-H]^-^ at m/z 783.5809 and trimmer [3M-H]^-^ at m/z 1175.8707. Positive ions polarity spectra (**C**,**D**) show protonated molecule [M+H]^+^ at m/z 393.3011, ammonium adduct [M+NH_3_]^+^ at m/z 410.3265, molecular acylium form [M-OH]^+^ at m/z 375.2900, molecular mono-dehydrated acylium form [M-H_2_O-OH]^+^ at m/z 357.2796 and molecular di-dehydrated acylium form [M-2H_2_O-OH]^+^ at m/z 339.2689.

**Fig. SI 17** HRMS-MS data analysis of Compound L (CoQ10 catabolite) at 5.1 min

MS data at 5.1 min of negative ions polarity (**A**) shows molecular formate adduct [M+formate-H]^-^ at m/z 459.2028. No attributed fragment was detected on fragmentation spectrum (**B**).

**Fig. SI 18** HRMS-MS data analysis of Compound M (CoQ10 catabolite) at 5.3 min

MS data at 5.3 min of negative ions polarity (**A**) shows deprotonated molecule [M-H]^-^ at m/z 547.2551. Fragmentation spectrum (**B**) shows *p*-quinol fragment at m/z 221.1202 and tail fragment at m/z 171.1024.

**Fig. SI 19** Folding of *h*COQ2 sequence using UbiA homolog from *Aeropyrum pernix* K1 (PDB 4OD5) as template. Parental crystallographic structure of 4OD5 (orange) contains PHBA (green), geranyl S-thiolodiphosphate (purple) and two Mg^2+^ (light green) as active subtracts. *h*COQ2 (light blue) presents 100% homology confidence, 96% sequence coverage and 23% sequence identity.

**Fig. SI 20** Detailed folding result obtained of Phyre2.2 server to *h*COQ2 sequence.

**Fig. SI 21** Docking results of PHBA (**a**) and MHQ-4-sulfate (**b**). Favorable binding orientation are bold highlighted.

**a**

**b**
